# Supplementary material for: Developing a clinical prediction rule for repeated consultations with functional somatic symptoms in primary care: a cohort study
Source: BMJ Open. 2021 Jan 8;11(1):e040730. doi: 10.1136/bmjopen-2020-040730 (PMC7799137; doi:10.1136/bmjopen-2020-040730)
Supplement: Supplementary data [file bmjopen-2020-040730supp001.pdf]

Supplemental Table 1. Functional somatic symptoms and the corresponding International Classification of Primary Care codes

**Symptom**	**International Classification of Primary Care code**
Generalized pain	A01
Fatigue/Tiredness/weakness	A04
Abdominal pain	D01
Flatulence	D08
Nauseous	D09
Constipation	D12
Defecation problems	D18
Irritable Bowel Syndrome	D93
Chest pain	K01; K02
Palpitations	K04
Joint pain	L01; L08
Back pain	L02; L03
Extremities pain	L09; L14
Headache	N01; N02
Wheezy	N02
Dizzy	N20
Sleep disorder	P06
Concentration problems	P20
Sore Throat	R21
Loss in appetite	T03
Weight gain/loss	T07; T08
