# Supplementary material for: Developing a clinical prediction rule for repeated consultations with functional somatic symptoms in primary care: a cohort study
Source: BMJ Open. 2021 Jan 8;11(1):e040730. doi: 10.1136/bmjopen-2020-040730 (PMC7799137; doi:10.1136/bmjopen-2020-040730)
Supplement: Supplementary data [file bmjopen-2020-040730supp002.pdf]

**Supplemental Table 2.** Comparison of patients with and without missing values

| | **No missing values (n = 2,002)** | | **Missing values (n = 648)** | | **Univariable OR (95% CI)** |
|---|---|---|---|---|---|
| | ***n*** | **value** | ***n*** | **value** | |
| Age, mean y (SD) | 2,002 | 42 (12) | 634 | 54 (17) | **1.07 (1.06–1.07)** |
| Male, n (%) | 2,002 | 622 (31) | 648 | 226 (35) | 1.19 (0.99–1.43) |
| Neuroticism, median (IQR) | 2,002 | 10.1 (9.1–11.3) | 246 | 10.3 (9.3–11.5) | 1.04 (0.96–1.12) |
| Chronic stress, median (IQR) | 2,002 | 3 (1–4) | 463 | 2 (0–4) | **0.90 (0.86–0.94)** |
| Stressful life events, median (IQR) | 2,002 | 1 (0–2) | 462 | 1 (0–2) | **1.09 (1.02–1.17)** |
| Self-rated health, median (IQR) | 2,002 | 3 (2–3) | 546 | 3 (2–3) | 1.10 (0.97–1.25) |
| Healthy activity[a], n (%) | 2,002 | 1,056 (53) | 272 | 203 (75) | **2.63 (1.98–3.51)** |
| Body mass index (kg/m$^2$), median (IQR) | 2,002 | 25 (23–28) | 646 | 26 (24–29) | **1.05 (1.03–1.07)** |
| Living alone, n (%) | 2,002 | 233 (12) | 520 | 102 (20) | **1.85 (1.43–2.39)** |
| Higher education[b], n (%) | 2,002 | 1,412 (71) | 570 | 295 (52) | **0.45 (0.37–0.54)** |
| MDD[c], n (%) | 2,002 | 64 (3) | 553 | 22 (4) | 1.25 (0.77–2.06) |
| GAD[d], n (%) | 2,002 | 138 (7) | 553 | 27 (5) | 0.69 (0.45–1.06) |
| Psychiatric consultations last year[e,f], n (%) | 2,002 | 205 (10) | 648 | 87 (13) | 1.07 (0.96–1.19) |
| GP consultations last year[f], median (IQR) | 2,002 | 2 (0–5) | 648 | 3 (1–6) | **1.05 (1.03–1.08)** |
| Repeated consultations[g], n (%) | 2,002 | 200 (10) | 648 | 97 (15) | **1.59 (1.22–2.06)** |

[a] Healthy activity, defined as 30 minutes at least 5 days a week.
[b] Higher education, defined as at least secondary vocational education or work-based training.
[c] MDD: Major Depressive Disorder.
[d] GAD: Generalized Anxiety Disorder.
[e] Patients with a consultation code in the P chapter of the International Classification of Primary Care.
[f] Predictors from NPCD and both are continuous variables. Other predictors are from Lifelines database.
[g] Repeated consultations is defined as ≥3 extra functional somatic symptoms consultations during one year of follow-up.
Note: 24% (648/2,650) had a missing value
Abbreviations: SD, standard deviation; IQR, Interquartile Range; GP, general practitioner; OR, odds ratio
