# Supplementary material for: Developing a clinical prediction rule for repeated consultations with functional somatic symptoms in primary care: a cohort study
Source: BMJ Open. 2021 Jan 8;11(1):e040730. doi: 10.1136/bmjopen-2020-040730 (PMC7799137; doi:10.1136/bmjopen-2020-040730)
Supplement: Supplementary data [file bmjopen-2020-040730supp004.pdf]

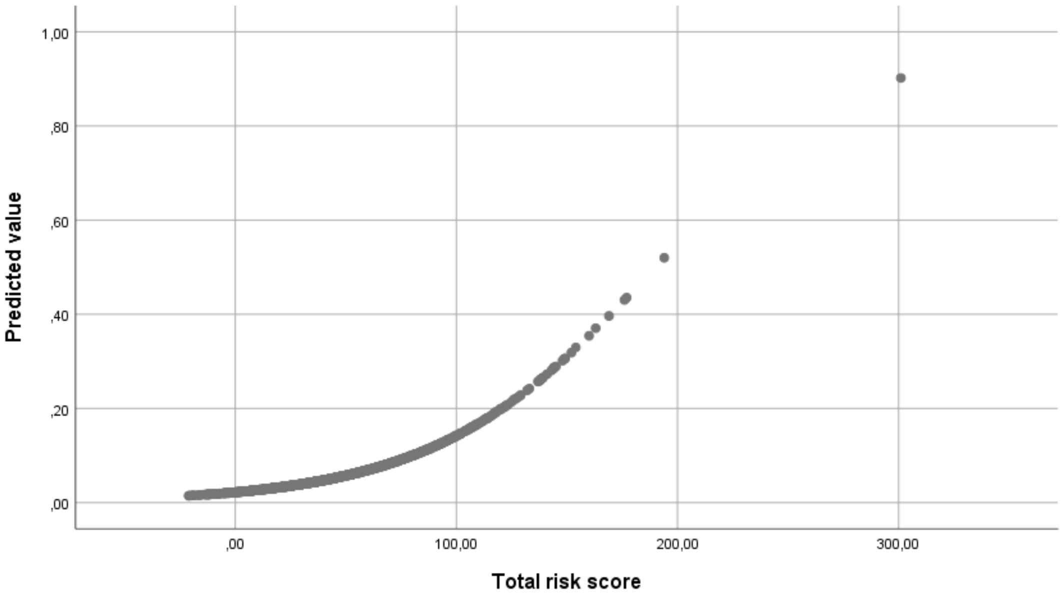

Supplemental Figure 2. Relation between the total risk score and the predicted risk of repeated consultations with functional somatic symptoms
