# Supplementary material for: Developing a clinical prediction rule for repeated consultations with functional somatic symptoms in primary care: a cohort study
Source: BMJ Open. 2021 Jan 8;11(1):e040730. doi: 10.1136/bmjopen-2020-040730 (PMC7799137; doi:10.1136/bmjopen-2020-040730)
Supplement: Author's manuscript [file bmjopen-2020-040730.draft_revisions.pdf]

BMJ Open

BMJ Open is committed to open peer review. As part of this commitment we make the peer review history of every article we publish publicly available.

When an article is published we post the peer reviewers' comments and the authors' responses online. We also post the versions of the paper that were used during peer review. These are the versions that the peer review comments apply to.

The versions of the paper that follow are the versions that were submitted during the peer review process. They are not the versions of record or the final published versions. They should not be cited or distributed as the published version of this manuscript.

BMJ Open is an open access journal and the full, final, typeset and author-corrected version of record of the manuscript is available on our site with no access controls, subscription charges or pay-per-view fees (http://bmjopen.bmj.com).

If you have any questions on BMJ Open's open peer review process please email info.bmjopen@bmj.com

BMJ Open

Developing a clinical prediction rule for frequent attenders with functional symptoms in primary care

Journal:	*BMJ Open*
Manuscript ID	bmjopen-2020-040730
Article Type:	Original research
Date Submitted by the Author:	20-May-2020
Complete List of Authors:	Holtman, Gea; UMCG, Department of general practice and elderly care medicine<br>Burger, H; UMCG<br>Verheij, Robert A; NIVEL, Netherlands Institute for Health Services Research<br>Wouters, Hans<br>Berger, Marjolein; UMCG, General Practice<br>Rosmalen, Judith; UMCG<br>Verhaak, Peter FM; UMCG
Keywords:	EPIDEMIOLOGY, PRIMARY CARE, GENERAL MEDICINE (see Internal Medicine)

SCHOLARONE™
Manuscripts

I, the Submitting Author has the right to grant and does grant on behalf of all authors of the Work (as defined in the below author licence), an exclusive licence and/or a non-exclusive licence for contributions from authors who are: i) UK Crown employees; ii) where BMJ has agreed a CC-BY licence shall apply, and/or iii) in accordance with the terms applicable for US Federal Government officers or employees acting as part of their official duties; on a worldwide, perpetual, irrevocable, royalty-free basis to BMJ Publishing Group Ltd ("BMJ") its licensees and where the relevant Journal is co-owned by BMJ to the co-owners of the Journal, to publish the Work in this journal and any other BMJ products and to exploit all rights, as set out in our licence.

The Submitting Author accepts and understands that any supply made under these terms is made by BMJ to the Submitting Author unless you are acting as an employee on behalf of your employer or a postgraduate student of an affiliated institution which is paying any applicable article publishing charge ("APC") for Open Access articles. Where the Submitting Author wishes to make the Work available on an Open Access basis (and intends to pay the relevant APC), the terms of reuse of such Open Access shall be governed by a Creative Commons licence – details of these licences and which Creative Commons licence will apply to this Work are set out in our licence referred to above.

Other than as permitted in any relevant BMJ Author's Self Archiving Policies, I confirm this Work has not been accepted for publication elsewhere, is not being considered for publication elsewhere and does not duplicate material already published. I confirm all authors consent to publication of this Work and authorise the granting of this licence.

For peer review only

Developing a clinical prediction rule for frequent attenders with functional symptoms in primary care

G.A. Holtman[1], H. Burger[1], R.A. Verheij[2,3], H. Wouters[1], M.Y. Berger[1], J.G.M. Rosmalen[4], P.F.M. Verhaak[1,2]

[1] University of Groningen, University Medical Center Groningen, Department of General Practice and Elderly Care Medicine, PO Box 196, 9700 AD Groningen, the Netherlands.

[2] Nivel, Netherlands Institute of Health Services Research, Utrecht, the Netherlands.

[3] Tilburg School of Social and Behavioral Sciences, Tranzo Scientific Center for Care and Welfare, Tilburg University, 90153 5000 LE Tilburg, The Netherlands.

[4] University of Groningen, University Medical Center Groningen, Interdisciplinary Center Psychopathology and Emotion Regulation, Groningen, the Netherlands.

Corresponding author: G.A. Holtman

Department of General Practice and Elderly Care Medicine,

University Medical Center Groningen,

University of Groningen,

PO Box 196, 9700 AD Groningen,

The Netherlands.

Telephone: 0031 642637125

E-mail address: g.a.holtman@umcg.nl

For peer review only

ABSTRACT

Objectives: Patients who present in primary care with chronic functional symptoms have reduced quality of life and increased health care costs. Recognizing these early is a challenge. The aim is to develop and internally validate a clinical prediction rule for frequent attenders with functional symptoms.

Design and setting: Records from the longitudinal population-based ("Lifelines") cohort study were linked to electronic health records from general practitioners (GPs).

Participants: We included patients consulting a GP with functional symptoms within one year after baseline assessment in the Lifelines cohort.

Outcome measures: The outcome is frequent attendance with functional symptoms, defined as ≥3 extra consultations after the first consultation. Multivariable logistic regression, with bootstrapping for internal validation, was used to develop a risk prediction model from 14 literature-based predictors. Model discrimination, calibration, and diagnostic accuracy were assessed.

Results: 18,810 participants were identified by database linkage, of whom 2,650 consulted a GP with functional symptoms and 297 (11%) attended frequently. In the final multivariable model, older age, female sex, lack of healthy activity, presence of generalized anxiety disorder, and higher number of GP consultations in the last year predicted frequent attendance. Discrimination after internal validation was 0.64 with a calibration slope of 0.95. The positive predictive value of patients with high scores on the model was 0.37 (0.29–0.47).

Conclusions: Several theoretically suggested predisposing and precipitating predictors, including neuroticism and stressful events, surprisingly failed to contribute to our final model. However, this model mostly included general predictors of increased risk of frequent attendance among patients with functional symptoms. Moreover, the model discrimination and positive predictive values were insufficient and preclude clinical implementation.

Keywords: medically unexplained symptoms, cohort studies, clinical decision rules, primary health care

For peer review only

Strengths and limitations of this study

- This study offers valuable insights into the predictors that could help general practitioners to identify frequent attenders with functional symptoms.
- By linking routine health care data from primary care to a large population-based cohort, we could include relevant predictors based on epidemiological and theoretical factors from the literature.
- Each patient had a full follow-up of 1 year.
- Time from baseline assessment of the population-based cohort to first GP consultation varied, however did this not affect the results.
- We did not externally validate the model, however the performance need to be improved before such research can be considered.

For peer review only

For peer review only - http://bmjopen.bmj.com/site/about/guidelines.xhtml

INTRODUCTION

Functional symptoms represent those that cannot be explained by a physical disease and account for about a third of all presentations in primary care,[1,2] clustering as cardiopulmonary, musculoskeletal, gastrointestinal, and general somatic symptoms.[3,4] However, these clusters appear to correlate and considered to represent one condition with different manifestations.[5] Most patients with functional symptoms consult a general practitioner (GP) only once, but 10%–30% of cases will become chronic,[6] leading to more diagnostic tests, more referrals, higher health care costs, and more psychological distress compared with other patients.[7–9] Recognizing those patients at risk of developing chronic symptoms and attending frequently could therefore help to target interventions that reduce symptom severity,[10,11] improve quality of life, and reduce GP workloads. Ensuring that these patients are identified early is an important challenge facing GPs,[12] and one for which a validated clinical prediction rule may help. Several factors are known to increase the risk of chronicity of functional symptoms, including predisposing (e.g., neuroticism), precipitating (e.g., physical and psychosocial stressors), and perpetuating (e.g., lack of healthy physical activity) factors.[13–15] Despite being described in the literature,[6] these factors have yet to be combined in a clinical prediction rule for use in primary care.

In this study, we aimed to develop and internally validate a clinical prediction rule for frequent attendance among patients who consult GPs with functional symptoms.

For peer review only

METHOD

Data sources

We linked patient records from the Lifelines Cohort Study ("Lifelines")[16] with those from the Nivel Primary Care Database (NPCD).[17] Dutch law conditionally allows the use of such electronic health records for research purposes. Statistics Netherlands (CBS) then used temporary record identification numbers to link records at an individual level for analysis.

For peer review only - http://bmjopen.bmj.com/site/about/guidelines.xhtml

Lifelines is a multidisciplinary prospective population-based cohort study using a three-generation design to examine the health and health-related behaviors of 167,729 people living in the north of the Netherlands.[16] It employs a broad range of investigative procedures to assess key factors that contribute to health and disease in the general population, focusing on multimorbidity and complex genetics. Lifelines was approved by the medical ethics committee of the University Medical Centre Groningen and was conducted in accordance with the Declaration of Helsinki. All participants signed an informed consent form.

The NPCD contains routinely recorded clinical data from GP consultations with patients, and is considered representative of the Dutch population.[17] The Dutch healthcare system is such that all non-institutionalized members of the population are registered with a general practice, which in turn, serves as a gatekeeping system through which patients must pass to access specialist care via GP referral.[18] In total, 528 general practices participated in 2019, and this study was approved according the Nivel Governance Code (number NZR0317.033).

For the current study, we included the baseline data of 152,728 adults enrolled in Lifelines between November 2006 and June 2013, and we linked these with the electronic health records of GP consultations for patients aged ≥18 years who consulted one of the 65 general practices in the north of the Netherlands that participated in the NPCD.

Patient population

We planned to include adults with functional symptoms considered at risk of becoming frequent attenders, which we defined as those having a GP consultation for a functional symptom in the year after their baseline assessment for Lifelines. The presence of functional symptoms was assessed based on the International Classification of Primary Care codes that related to the symptoms that Robbins et al. described (see Supplementary Table 1).[19]

Outcomes

The primary outcome was frequent attendance with functional symptoms, defined as ≥3 extra GP

For peer review only - http://bmjopen.bmj.com/site/about/guidelines.xhtml

consultations for one of the defined functional symptoms (Supplementary Table 1) during a year of follow-up after first consulting a GP with that symptom.[19,20] Complete follow-up data were recorded for all GP consultations in electronic health records, and we permitted the functional symptoms to vary between consultations.

Candidate predictors

We selected 14 predictors based on literature review and expert opinion: age, sex, neuroticism, chronic stress, life events, quality of life, physical activity, body mass index, living alone, education, major depressive disorder (MDD), generalized anxiety disorder (GAD), and psychiatric or GP consultations in the 12 months before first consulting with functional symptoms.[6,21] The data for these predictors were derived from the baseline of Lifelines, except for the psychiatric and GP consultations, which were derived from the NPCD.

Neuroticism was evaluated using an abridged version of the Neuroticism Extraversion Openness–Personality Inventory–Revised that included only anger-hostility, self-consciousness, impulsivity, and vulnerability, and excluded depression and anxiety (score range, 4–32).[22] Chronic stress was measured with the Long-term Difficulties Inventory (score range, 0–24).[23] The List of Threatening Events was used to assess the occurrence of 12 stressful life events (score range, 0–12).[23,24] Quality of life was evaluated with the RAND-36 question[25] "how would you rate your health from 1 (excellent) to 5 (poor)." The Short Questionnaire to Assess Health-Enhancing Physical Activity was used to determine physical activity behavior, with a cut-off of 30 minutes at least 5 days a week indicating healthy activity.[26] Higher education was defined as at least secondary vocational education or work-based training. MDD and GAD were assessed by the Mini-International Neuropsychiatric Interview, compatible with International Classification of Disease, Tenth Edition, and the Diagnostic and Statistical Manual of Mental Disorders, Fourth Edition.[27]

Sample size

We estimated that we required 11,455 participants based on an assumption that 10% of the NPCD

For peer review only - http://bmjopen.bmj.com/site/about/guidelines.xhtml

cohort would participate in Lifelines and that 75% of these data could be linked (i.e., 10% × 75% × 152,728). Given that the prevalence of frequent attendance with functional symptoms has been reported to be 2.5%, we estimated that 286 of these could be included[20] to achieve an effective sample size of at least 20 outcome events per predictor.[28]

Missing data

Eleven predictors from Lifelines had missing data, so we evaluated the underlying causes and patterns to assess the need for multiple imputation.[29] When appropriate, we replaced all missing values by chained equations, incorporating all variables used in the analyses, including the outcome variable, and all variables that predicted missingness of a certain variable or value. We imputed questionnaire sum scores rather than item scores. Finally, we constructed 20 imputed datasets combined across all datasets, pooled β coefficients, and calculated odds ratios using Rubin's rule.[30]

Statistical analysis

Frequent attendance with functional symptoms over a one-year follow-up period was set as the binary outcome variable and associated with potential predictors as independent variables in logistic regression analyses. We performed univariable analyses to calculate unadjusted odds ratios.

To develop the clinical prediction rule, we initially included all potential predictors in a multivariable logistic regression model, irrespective of their univariable association and refrained from univariable preselection of candidate predictors to prevent model instability.[31] Using backward stepwise selection, we excluded predictors from the model that were not statistically significant according to Akaike's information criterion (i.e., $p > 0.157$) in >50% of all imputed datasets.[32] Time between baseline assessment of predictors and first consultation differed between participants, so we also evaluated its influence in a separate analysis. We assessed rule performance by its discriminatory power with the C statistic and the calibration slope. We internally validated the model to correct for over-optimism by bootstrapping 250 samples, calculating a shrinkage factor, multiplying the original β coefficients by this factor, and re-estimating the intercepts using the shrunken β coefficients. The β

For peer review only - http://bmjopen.bmj.com/site/about/guidelines.xhtml

coefficients were translated into a risk score of whole numbers for ease of use by GPs when evaluating the risk of frequent attendance in clinical practice. To that end, each β coefficient was divided by the coefficient closest to zero and then rounded to the nearest integer. The total score for each patient was calculated as the sum of all points for each predictor. We calculated the sensitivity, specificity, and positive predictive value of the rule at several thresholds to distinguish high and low risk. Thresholds were chosen arbitrarily based on the sample sizes being adequate in each category and the clinical risk being distinguishable.

All statistical analyses were performed with STATA/SE15 (STATA Corp, College station, TX, USA) and R (for bootstrapping). The Transparent Reporting of a multivariable prediction model for Individual Prediction of Diagnosis (TRIPOD) was used to conduct this study and report its results.[33]

RESULTS

Study participants

Of the 152,728 Lifelines participants with a baseline assessment, we linked 18,810 (12%) with NPCD data (Figure 1). Among these, we included 2,650 participants (14% of those linked) attending GP consultations for a functional symptoms (i.e., the at-risk group), of whom 297 (11%) had ≥3 further consultations for functional symptoms (i.e., the outcome criterion). The details of the included and excluded patients are summarized in Table 1, showing that the groups were broadly comparable. Notably, 24% of participants had a missing value and 3% had missing values for >4 predictors. The participants with missing values were slightly older and less active, and they less often had completed higher education (Supplementary Table 2).

Clinical rule development

Univariable associations of the potential predictors for frequent attendance with functional symptoms are listed in Supplementary Table 3. In the final multivariable model, the following five

For peer review only - http://bmjopen.bmj.com/site/about/guidelines.xhtml

predictors were selected based on significant associations: higher age, female sex, lack of healthy activity, presence of GAD, and having had more GP consultations in the year before first consulting with functional symptoms (Table 2). Time from baseline to first consultation did not affect the results. The shrinkage factor of 0.95 showed limited model overfitting and was applied to adjust predictor coefficients in the final model. Likewise, the C statistic (area under the curve) of 0.65 (95% CI, 0.62–0.69) was corrected to 0.64 (95% CI, 0.61–0.68). Agreement between the observed and predicted proportion of events showed adequate calibration (Supplementary Figure 1).

The final model could calculate the absolute predicted individual risk of frequent attendance with functional symptoms (Figure 2). For a risk score ≥100, the positive predictive value of frequent attendance was 0.37 (95% CI, 0.29–0.47) (Tables 3 and 4). However, when increasing the cut-off from 25 to 100, the sensitivity decreased from 0.87 (95% CI, 0.83–0.91) to 0.13 (95% CI, 0.10–0.17) and the specificity increased from 0.23 (95% CI, 0.22–0.25) to 0.97 (95% CI, 0.97–0.98).

DISCUSSION

Summary

We developed and internally validated a clinical prediction rule to identify patients at high risk of frequent attendance with functional symptoms. This was based on five factors that are readily available in primary care: age, sex, activity levels, GAD diagnosis, and number of consultations. However, despite being well calibrated, the prediction rule showed poor discrimination. Nevertheless, if patients scored ≥100, the risk of frequent attendance with functional symptoms increased to 37% from the baseline value of 11%.

Strengths and limitations

The study benefited from the use of a rich data set established by linking routine electronic health record data from primary care to a large population-based cohort. We effectively linked 18,810

For peer review only

patients (12%) from Lifelines who had at least one GP consultation, and we could include predictors based on epidemiological and theoretical factors from the literature, such as neuroticism and threatening events.[21] The data linkage approach that we adopted may serve to enhance primary care research in the future. Each patient also had follow-up data for a full year, and although the time from baseline assessment in Lifelines to first GP consultation varied because of the dynamic nature of the NPCD cohort, this did not affect the results. Another strength is that we included 21 events per variable, resulting in minimal overfitting with a shrinkage factor of 0.95. An advantage of using dichotomous over continuous outcomes is that clinical interpretation is more straightforward. Although it is problematic that we did not externally validate the model, we contend that the model's performance will need to be improved before such research can be considered.

Our model predicts the risk of having ≥3 extra consultations for functional symptoms. However, this should not be confused with predicting a functional somatic syndrome, not least because we could not determine this diagnosis with the available data. A disadvantage of our outcome measure is that patients with functional symptoms may also have consulted other health care professionals (e.g., physiotherapist), so these cases may have been missed. Our approach to identify the at-risk population first may explain the contrast with existing data. For example, we showed that 11% of patients presenting with functional symptoms ultimately had ≥4 consultations for these symptoms, whereas previous research has shown a rate of 2.5% among all patients with GP consultations.[20]

Comparison with other studies

We are aware of no other clinical prediction rules for frequent attenders consulting their GP with functional symptoms. It should be emphasized that such a model cannot be considered synonymous with explaining the cause.[34] We found two studies that developed models by combining predictors using a backward selection procedure.

One study used information from GP letters to medical specialists for patients who were referred with functional somatic symptoms.[35] In their clinical prediction rule, female sex, referral symptom

For peer review only - http://bmjopen.bmj.com/site/about/guidelines.xhtml

group, lack of somatic comorbidity, lack of abnormal physical findings, history of psychiatric diagnosis or treatment, and referral letter written in illness terminology were all shown to be predictors. This model had a higher area under the curve (0.80) than ours (0.64) and was developed for patients consulting internists. However, the GP referral letters included relevant predictors that helped to identify functional somatic symptoms, and although the population was more selected than ours, the results show that data collected in primary care can be suitable predictors.

The other study developed a model for symptom severity and for both physical and mental functioning during a 2-year follow-up period among patients with functional symptoms.[21] They predicted severe courses by physical comorbidity, higher baseline severity and longer functional symptom duration, anxiety, catastrophizing cognitions, embarrassment, and neuroticism, as well as fear avoidance, avoidance, or resting behavior. By contrast, they predicted favorable courses based on limited alcohol use, higher education, higher baseline physical and mental functioning, symptom focusing, damage cognitions, and extraversion. Although we also identified anxiety as a predictor, we did not find the same for neuroticism or higher education. Also contrasting with our data, as well as that of others,[36,37] they did not show that female sex was a predictor. Unfortunately, we could not include predictors of illness cognitions because these were not evaluated in Lifelines. Indeed, the Symptoms Checklist 90 questionnaire had more than 50% missing values during baseline evaluation in Lifelines, so we excluded these data.[16] The differences in identified predictors may be explained by different study populations, predictor selection criteria, or outcomes.

Implications for research and practice

To our surprise, several theoretically suggested predisposing and precipitating predictors, including neuroticism and stressful events, failed to contribute to the final prediction model. Instead, this model included mostly general predictors that provide little additional information to help GPs recognize patients at risk of becoming frequent attenders with functional symptoms, and it not only has poor discrimination and positive predictive value but also lacks external validation. Therefore, at

For peer review only - http://bmjopen.bmj.com/site/about/guidelines.xhtml

present, we cannot recommend the score for clinical use. Nevertheless, our findings indicate that GPs might expect chronicity when older women with low activity levels and anxiety symptoms present with functional symptoms. These require extra vigilance and may benefit from early intervention with self-help advice.[11] Some predictors identified in earlier studies, such as female sex and anxiety, could be potential factors in future clinical prediction rules designed to help GPs recognize patients at risk of becoming a frequent attender.

For peer review only

For peer review only - http://bmjopen.bmj.com/site/about/guidelines.xhtml

FUNDING

This study was funded by a grant from the Healthy Ageing Pilot of the University Medical Center Groningen. The Lifelines initiative has been made possible by support from the Dutch Ministry of Health, Welfare and Sport; the Dutch Ministry of Economic Affairs; University Medical Center Groningen; Groningen University; and the Provinces in the north of the Netherlands (Drenthe, Friesland, Groningen).

ETHICAL APPROVAL

The medical ethics committee of the University Medical Centre Groningen approved the Lifelines study. Dutch law conditionally allows the use of electronic health records for research purposes.

COMPETING INTERESTS

None declared.

PATIENT CONSENT FORM

Not required.

AUTHOR CONTRIBUTORS:

Study concept and design: GAH, HB, PFMV. Acquisition of data: PFMV and RAV. Analysis of data: GAH. Interpretation of data: GAH, HB, RAV, HW, MYB, JGMR, PFMV. Drafting the work: GAH, HB, PFMV. The final manuscript was critically revised and approved by all authors.

DATA AVAILABILITY STATEMENT:

The data used is part of two ongoing databases: Lifelines Cohort Study and Nivel Primary Care Database. The data is not available. The corresponding author can be contacted for details.

WORD COUNT

2556 words

ACKNOWLEDGMENT

For peer review only

We thank Dr Robert Sykes (www.doctored.org.uk) for providing editorial services.

For peer review only

For peer review only - http://bmjopen.bmj.com/site/about/guidelines.xhtml

REFERENCES

1. Jackson JL, Passamonti M. The outcomes among patients presenting in primary care with a physical symptom at 5 years. *Journal of general internal medicine*. 2005;20(11):1032-1037.

2. Rosendal M, Carlsen AH, Rask MT, Moth G. Symptoms as the main problem in primary care: A cross-sectional study of frequency and characteristics. *Scand J Prim Health Care*. 2015;33(2):91-99.

3. Fink P, Toft T, Hansen MS, Ornbol E, Olesen F. Symptoms and syndromes of bodily distress: An exploratory study of 978 internal medical, neurological, and primary care patients. *Psychosom Med*. 2007;69(1):30-39.

4. Rosmalen J, Tak L, De Jonge P. Empirical foundations for the diagnosis of somatization: Implications for DSM-5. *Psychol Med*. 2011;41(6):1133-1142.

5. Henningsen P, Zipfel S, Sattel H, Creed F. Management of functional somatic syndromes and bodily distress. *Psychother Psychosom*. 2018;87(1):12-31.

6. olde Hartman TC, Borghuis MS, Lucassen PL, van de Laar, Floris A, Speckens AE, van Weel C. Medically unexplained symptoms, somatisation disorder and hypochondriasis: Course and prognosis. A systematic review. *J Psychosom Res*. 2009;66(5):363-377.

7. Dirkzwager AJ, Verhaak PF. Patients with persistent medically unexplained symptoms in general practice: Characteristics and quality of care. *BMC Family Practice*. 2007;8(1):33.

8. Burton C, McGorm K, Richardson G, Weller D, Sharpe M. Healthcare costs incurred by patients repeatedly referred to secondary medical care with medically unexplained symptoms: A cost of illness study. *J Psychosom Res*. 2012;72(3):242-247.

9. De Waal, Margot W M, Arnold IA, Eekhof JA, Van Hemert AM. Somatoform disorders in general practice: Prevalence, functional impairment and comorbidity with anxiety and depressive disorders. *The British Journal of Psychiatry*. 2004;184(6):470-476.

10. Van Dessel N, Den Boeft M, van der Wouden, Johannes C, et al. Non-pharmacological interventions for somatoform disorders and medically unexplained physical symptoms (MUPS) in adults. *Cochrane Database of Systematic Reviews*. 2014(11).

For peer review only

11. van Gils A, Schoevers RA, Bonvanie IJ, Gelauff JM, Roest AM, Rosmalen JG. Self-help for medically unexplained symptoms: A systematic review and meta-analysis. *Psychosom Med*. 2016;78(6):728-739.

12. Rosendal M, Hartman TCO, Aamland A, et al. Medically unexplained symptoms and symptom disorders in primary care: Prognosis-based recognition and classification. *BMC family practice*. 2017;18(1):18.

13. Frølund Pedersen H, Frostholm L, Søndergaard Jensen J, Ørnbøl E, Schröder A. Neuroticism and maladaptive coping in patients with functional somatic syndromes. *British journal of health psychology*. 2016;21(4):917-936.

14. Salit IE. Precipitating factors for the chronic fatigue syndrome. *J Psychiatr Res*. 1997;31(1):59-65.

15. Van Houdenhove B, Luyten P. Customizing treatment of chronic fatigue syndrome and fibromyalgia: The role of perpetuating factors. *Psychosomatics*. 2008;49(6):470-477.

16. Scholtens S, Smidt N, Swertz MA, et al. Cohort profile: LifeLines, a three-generation cohort study and biobank. *Int J Epidemiol*. 2014;44(4):1172-1180.

17. NIVEL. Https://Www.nivel.nl/nl/nivel-zorgregistraties-eerste-lijn/nivel-zorgregistraties-eerste-lijn. https://www.nivel.nl/nl/nivel-zorgregistraties-eerste-lijn/nivel-zorgregistraties-eerste-lijn.

18. Linden M, Gothe H, Ormel J. Pathways to care and psychological problems of general practice patients in a “gate keeper” and an” open access” health care system. *Soc Psychiatry Psychiatr Epidemiol*. 2003;38(12):690-697.

19. Robbins JM, Kirmayer LJ, Hemami S. Latent variable models of functional somatic distress. *J Nerv Ment Dis*. 1997;185(10):606-615.

20. Verhaak PF, Meijer SA, Visser AP, Wolters G. Persistent presentation of medically unexplained symptoms in general practice. *Fam Pract*. 2006;23(4):414-420.

21. Claassen-van Dessel N, van der Wouden, Johannes C, Twisk JW, Dekker J, van der Horst, Henriëtte E. Predicting the course of persistent physical symptoms: Development and internal validation of prediction models for symptom severity and functional status during 2 years of follow-

For peer review only - http://bmjopen.bmj.com/site/about/guidelines.xhtml

up. *J Psychosom Res*. 2018;108:1-13.

22. Costa PT, Mac Crae RR. *Neo personality inventory-revised (NEO PI-R).* Psychological Assessment Resources Odessa, FL; 1992.

23. Rosmalen J, Bos E, De Jonge P. Validation of the long-term difficulties inventory (LDI) and the list of threatening experiences (LTE) as measures of stress in epidemiological population-based cohort studies. *Psychol Med*. 2012;42(12):2599-2608.

24. Brugha TS, Cragg D. The list of threatening experiences: The reliability and validity of a brief life events questionnaire. *Acta Psychiatr Scand*. 1990;82(1):77-81.

25. Hays RD, Morales LS. The RAND-36 measure of health-related quality of life. *Ann Med*. 2001;33(5):350-357.

26. Wendel-Vos GW, Schuit AJ, Saris WH, Kromhout D. Reproducibility and relative validity of the short questionnaire to assess health-enhancing physical activity. *J Clin Epidemiol*. 2003;56(12):1163-1169.

27. Sheehan DV, Lecrubier Y, Sheehan KH, et al. The mini-international neuropsychiatric interview (MINI): The development and validation of a structured diagnostic psychiatric interview for DSM-IV and ICD-10. *J Clin Psychiatry*. 1998.

28. Peduzzi P, Concato J, Kemper E, Holford TR, Feinstein AR. A simulation study of the number of events per variable in logistic regression analysis. *J Clin Epidemiol*. 1996;49(12):1373-1379.

29. Hughes RA, Heron J, Sterne JA, Tilling K. Accounting for missing data in statistical analyses: Multiple imputation is not always the answer. *Int J Epidemiol*. 2019;1:11.

30. Marshall A, Altman DG, Holder RL, Royston P. Combining estimates of interest in prognostic modelling studies after multiple imputation: Current practice and guidelines. *BMC Med Res Methodol*. 2009;9:57-2288-9-57.

31. Harrell Jr FE, Lee KL, Mark DB. Multivariable prognostic models: Issues in developing models, evaluating assumptions and adequacy, and measuring and reducing errors. *Stat Med*. 1996;15(4):361-387.

For peer review only

32. Sauerbrei W. The use of resampling methods to simplify regression models in medical statistics. *Journal of the Royal Statistical Society: Series C (Applied Statistics)*. 1999;48(3):313-329.

33. Collins GS, Reitsma JB, Altman DG, Moons KG. Transparent reporting of a multivariable prediction model for individual prognosis or diagnosis (TRIPOD): The TRIPOD statement. *BMC medicine*. 2015;13(1):1.

34. Moons KG, Royston P, Vergouwe Y, Grobbee DE, Altman DG. Prognosis and prognostic research: What, why, and how? *BMJ*. 2009;338:b375.

35. Gol JM, Burger H, Janssens KA, Slaets JP, Gans RO, Rosmalen JG. PROFSS: A screening tool for early identification of functional somatic symptoms. *J Psychosom Res*. 2014;77(6):504-509.

36. Speckens A, Van Hemert A, Bolk J, Rooijmans H, Hengeveld M. Unexplained physical symptoms: Outcome, utilization of medical care and associated factors. *Psychol Med*. 1996;26(4):745-752.

37. Leiknes KA, Finset A, Moum T, Sandanger I. Methodological issues concerning lifetime medically unexplained and medically explained symptoms of the composite international diagnostic interview: A prospective 11-year follow-up study. *J Psychosom Res*. 2006;61(2):169-179.

For peer review only

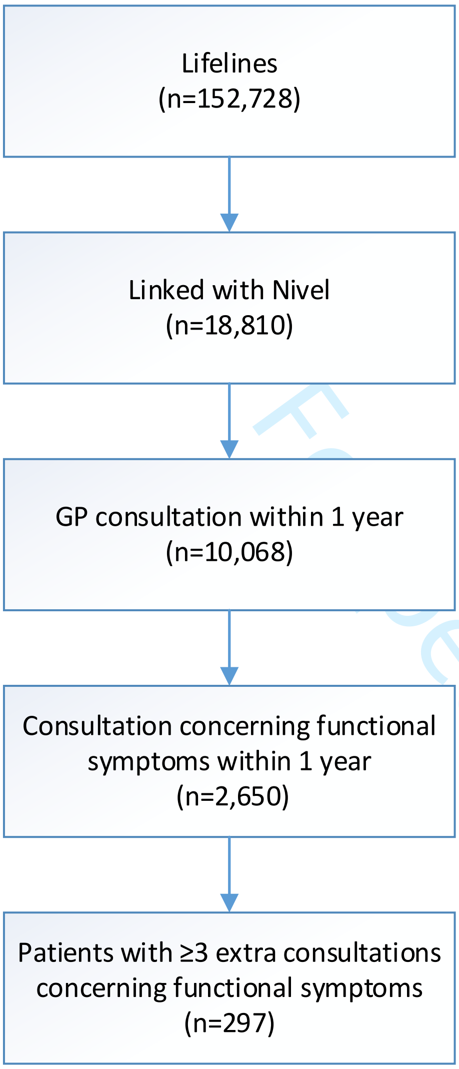

Figure 1. Patient selection flowchart

Note: extra consultations (≥3) refers to additional presentations for functional symptoms during a one-year follow-up period after an initial GP consultation for a functional symptom.

For peer review only - http://bmjopen.bmj.com/site/about/guidelines.xhtml

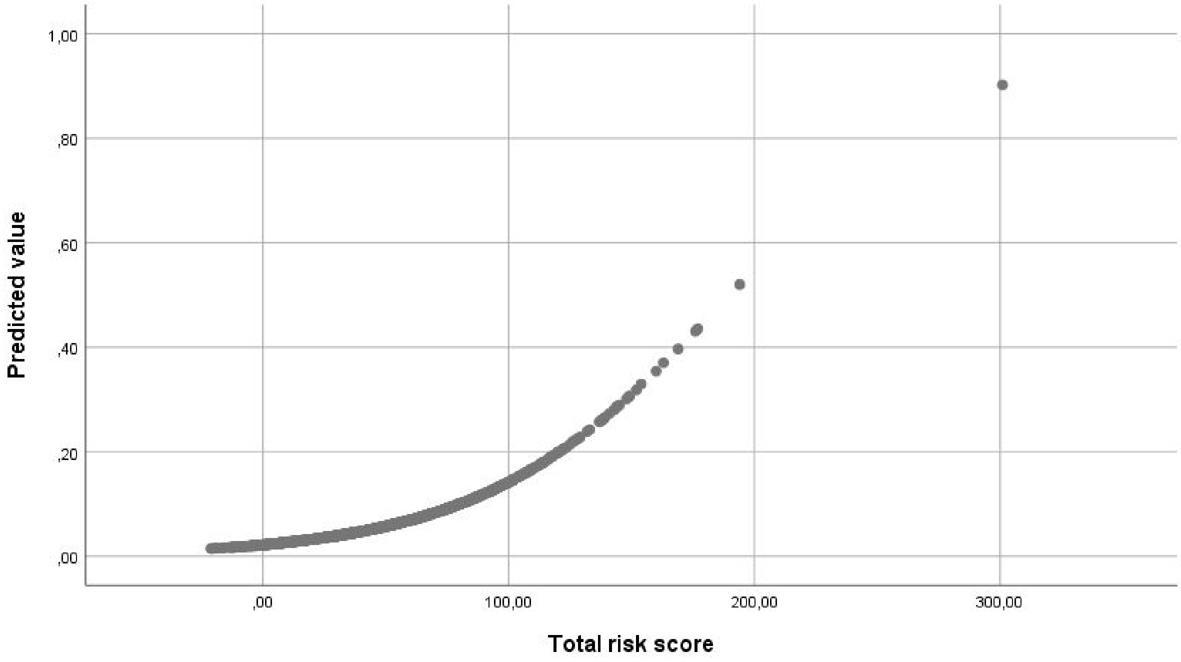

Figure 2. Relation between the total risk score and the predicted risk of frequent attendance with functional symptoms

For peer review only - http://bmjopen.bmj.com/site/about/guidelines.xhtml

Table 1. Characteristics of included and excluded patients

	Included patients[a] (n = 2,650)		Excluded patients[b] (n = 7,418)	
	N	Score	*n*	Score
Age, mean y (SD)	2,636	45 (14)	7,387	45 (14)
Female, n (%)	2,650	1,802 (68)	7,418	4,577 (62)
Neuroticism, median (IQR)	2,248	10.1 (9.1-11.3)	6,419	9.9 (8.9 – 11)
Chronic stress, median (IQR)	2,465	2 (1–4)	7,005	2 (1–4)
Threatening events, median (IQR)	2,464	1 (0–2)	7,008	1 (0–2)
Quality of Life, median (IQR)	2,548	3 (2–3)	7,228	3 (2–3)
Healthy activity[c], n (%)	2,274	1,259 (55)	6,505	3,589 (55)
Body mass index (kg/m^2), median (IQR)	2,648	26 (23–28)	7,417	25 (23–28)
Living alone, n (%)	2,522	335 (13.3)	7,167	904 (12.6)
Higher education[d], n (%)	2,572	1,707 (66)	7,215	5,067 (70)
MDD[f], n (%)	2,555	86 (3)	7,248	176 (2.4)
GAD[g], n (%)	2,555	165 (6)	7,248	351 (4.8)
Psychiatric consultations last year[h,i], n (%)	2,650	292 (11)	7,418	783 (11)
GP consultations last year[i], median (IQR)	2,650	2 (0–5)	7,418	1 (0–3)

[a] Included: GP consultations and ≥1 functional symptom within 1 year after baseline Lifelines assessment.

[b] Excluded: GP consultation without a functional symptom within 1 year after baseline of Lifelines.

[c] Healthy activity, defined as 30 minutes at least 5 days a week.

[d] Higher education, defined as at least secondary vocational education or work-based training.

[f] MDD: Major Depressive Disorder.

[g] GAD: Generalized Anxiety Disorder.

[h] Patients with a consultation code in the P chapter of the International Classification of Primary Care.

[i] Predictors from NPCD. Other predictors are from Lifelines.

For peer review only

Table 2. Final multivariable analysis for frequent attendance with functional symptoms

Predictors	OR (95% CI) [c]	P-value	Coefficient	Adjusted coefficient	Risk score
Constant	0.05 (0.03–0.08)	0.000	-2.95	-3.80	
Age	1.02 (1.01–1.03)	0.000	0.02	0.02	1
Sex (m)	0.75 (0.56–0.99)	0.042	-0.30	-0.29	-15
Healthy activity[a]	0.60 (0.45–0.80)	0.001	-0.51	-0.48	-24
GAD[b]	1.79 (1.17–2.74)	0.008	0.58	0.56	28
GP consultations last year	1.10 (1.07–1.14)	0.000	0.10	0.10	5

[a] Healthy activity, defined as 30 minutes at least 5 days a week.

[b] GAD: Generalized Anxiety Disorder.

[c] OR (95% CI): Odds Ratio (95% confidence interval).

Note: Shrinkage factor 0.96; predictors selected if P < 0.157.

For peer review only

For peer review only - http://bmjopen.bmj.com/site/about/guidelines.xhtml

Table 3. Risk of frequent attendance with functional symptoms by different cut-off scores

Cut-off score	*n*	Outcome	Observed risk	Predicted risk
<25	585	38	0.07	0.06
25–49	1009	82	0.08	0.08
50–99	952	138	0.15	0.14
≥100	104	39	0.38	0.37

Note: the risk score was calculated by multiplying each risk score by the predictor value, with the total score ranging from −21 to 301 for all included patients.

For peer review only

Table 4. Diagnostic accuracy of the risk score for frequent attendance with functional symptoms

Cut-off score	*n*	Sensitivity (95% CI) [c]	Specificity (95% CI) [c]	PPV[a] (95% CI) [c]	NPV[b] (95% CI) [c]
≥25	2065	0.87 (0.83–0.91)	0.23 (0.22–0.25)	0.13 (0.11–0.14)	0.93 (0.91–0.95)
≥50	1057	0.59 (0.54–0.65)	0.63 (0.61–0.64)	0.17 (0.15–0.19)	0.92 (0.91–0.94)
≥100	104	0.13 (0.10–0.17)	0.97 (0.97–0.98)	0.37 (0.29–0.47)	0.91 (0.90–0.92)

[a] PPV: positive predictive value.

[b] NPV: negative predictive value.

[c] 95% CI: 95% confidence interval.

For peer review only

Supplementary Table 1. Functional symptoms and the corresponding International Classification of Primary Care codes

Symptom	International Classification of Primary Care code
Generalized pain	A01
Fatigue/Tiredness/weakness	A04
Abdominal pain	D01
Flatulence	D08
Nauseous	D09
Constipation	D12
Defecation problems	D18
Irritable Bowel Syndrome	D93
Chest pain	K01; K02
Palpitations	K04
Joint pain	L01; L08
Back pain	L02; L03
Extremities pain	L09; L14
Headache	N01; N02
Wheezy	N02
Dizzy	N20
Sleep disorder	P06
Concentration problems	P20
Sore Throat	R21
Loss in appetite	T03
Weight gain/loss	T07; T08

For peer review only

Supplementary Table 2. Comparison of patients with and without missing values

	No missing values (n = 2,002)		Missing values (n = 648)		Univariable OR (95% CI)
	n	value	*n*	value	
Age, mean y (SD)	2,002	42 (12)	634	54 (17)	**1.07 (1.06–1.07)**
Male, n (%)	2,002	622 (31)	648	226 (35)	1.19 (0.99–1.43)
Neuroticism, median (IQR)	2,002	10.1 (9.1–11.3)	246	10.3 (9.3–11.5)	1.04 (0.96–1.12)
Chronic stress, median (IQR)	2,002	3 (1–4)	463	2 (0–4)	**0.90 (0.86–0.94)**
Threatening events, median (IQR)	2,002	1 (0–2)	462	1 (0–2)	**1.09 (1.02–1.17)**
Quality of Life, median (IQR)	2,002	3 (2–3)	546	3 (2–3)	1.10 (0.97–1.25)
Healthy activity[a], n (%)	2,002	1,056 (53)	272	203 (75)	**2.63 (1.98–3.51)**
Body mass index (kg/m^2), median (IQR)	2,002	25 (23–28)	646	26 (24–29)	**1.05 (1.03–1.07)**
Living alone, n (%)	2,002	233 (12)	520	102 (20)	**1.85 (1.43–2.39)**
Higher education[b], n (%)	2,002	1,412 (71)	570	295 (52)	**0.45 (0.37–0.54)**
MDD[c], n (%)	2,002	64 (3)	553	22 (4)	1.25 (0.77–2.06)
GAD[d], n (%)	2,002	138 (7)	553	27 (5)	0.69 (0.45–1.06)
Psychiatric consultations last year[e,f], n (%)	2,002	205 (10)	648	87 (13)	1.07 (0.96–1.19)
GP consultations last year[f], median (IQR)	2,002	2 (0–5)	648	3 (1–6)	**1.05 (1.03–1.08)**
Frequent attender[g], n (%)	2,002	200 (10)	648	97 (15)	**1.59 (1.22–2.06)**

[a] Healthy activity, defined as 30 minutes at least 5 days a week.

[b] Higher education, defined as at least secondary vocational education or work-based training.

[c] MDD: Major Depressive Disorder.

[d] GAD: Generalized Anxiety Disorder.

[e] Patients with a consultation code in the P chapter of the International Classification of Primary Care.

[f] Predictors from NPCD and both are continuous variables. Other predictors are from Lifelines database.

[g] Frequent attender is a patient with ≥3 extra functional symptom consultations during one year of follow-up.

For peer review only

Supplementary Table 3. Univariable analysis of predictors for frequent attendance with functional symptoms

Variable	Frequent attendance[a] OR (95% CI)
Age	1.01 (1.00–1.02)
Sex (m)	0.69 (0.52–0.91)
Neuroticism	1.08 (0.99–1.17)
Chronic stress	1.04 (0.99–1.09)
Threatening events	1.08 (0.99–1.18)
Quality of Life	1.41 (1.20–1.66)
Healthy activity[b]	0.70 (0.53–0.91)
Body mass index (kg/m^2)	1.04 (1.01–1.07)
Living alone	0.91 (0.62–1.32)
Higher education[c]	0.75 (0.58–0.96)
MDD[d]	1.53 (0.85–2.73)
GAD[e]	2.15 (1.45–3.19)
Psychiatric consultations last year[f,g]	1.17 (1.04–1.33)
GP consultations last year[g]	1.12 (1.09–1.15)

[a] Outcome, ≥3 extra functional symptom consultations during a 1-year follow-up period (n = 297).

[b] Healthy activity, defined as 30 minutes at least 5 days a week.

[c] Higher education, defined as at least secondary vocational education or work-based training.

[d] MDD: Major Depressive Disorder.

[e] GAD: Generalized Anxiety Disorder.

[f] Number of consultations concerning International Classification of Primary Care codes in the P chapter.

[g] Predictors are from NPCD and are continuous. Other predictors are from the Lifelines database.

For peer review only

Supplementary Figure 1. Calibration plot of the clinical prediction rule for frequent attendance with functional symptoms

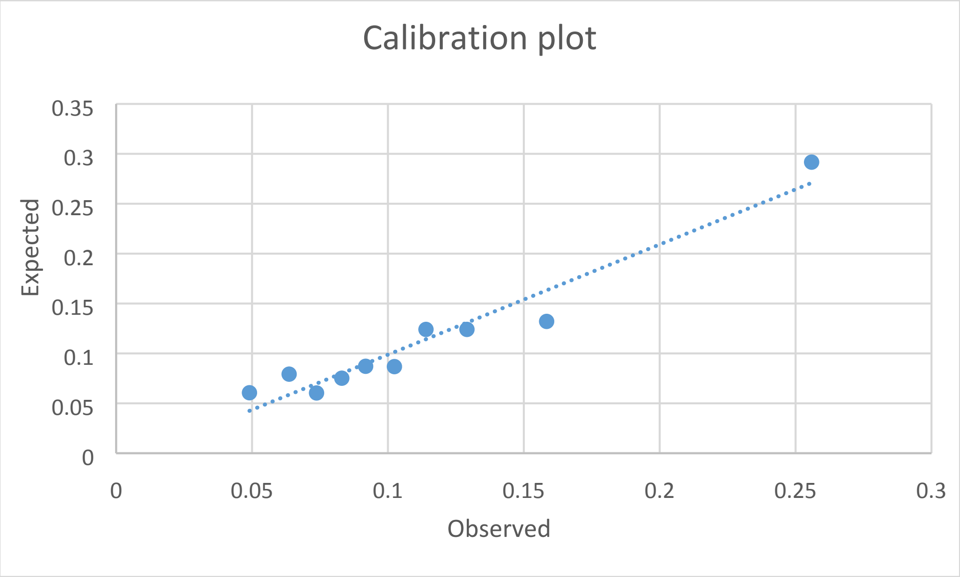

For peer review only - http://bmjopen.bmj.com/site/about/guidelines.xhtml

TRIPOD Checklist: Prediction Model Development

Section/Topic	Item	Checklist Item	Page
**Title and abstract**			
Title	1	Identify the study as developing and/or validating a multivariable prediction model, the target population, and the outcome to be predicted.	1
Abstract	2	Provide a summary of objectives, study design, setting, participants, sample size, predictors, outcome, statistical analysis, results, and conclusions.	2
**Introduction**			
Background and objectives	3a	Explain the medical context (including whether diagnostic or prognostic) and rationale for developing or validating the multivariable prediction model, including references to existing models.	3
	3b	Specify the objectives, including whether the study describes the development or validation of the model or both.	3
**Methods**			
Source of data	4a	Describe the study design or source of data (e.g., randomized trial, cohort, or registry data), separately for the development and validation data sets, if applicable.	4,5
	4b	Specify the key study dates, including start of accrual; end of accrual; and, if applicable, end of follow-up.	5,6
Participants	5a	Specify key elements of the study setting (e.g., primary care, secondary care, general population) including number and location of centres.	5
	5b	Describe eligibility criteria for participants.	5
	5c	Give details of treatments received, if relevant.	NA
Outcome	6a	Clearly define the outcome that is predicted by the prediction model, including how and when assessed.	5,6
	6b	Report any actions to blind assessment of the outcome to be predicted.	NA
Predictors	7a	Clearly define all predictors used in developing or validating the multivariable prediction model, including how and when they were measured.	6
	7b	Report any actions to blind assessment of predictors for the outcome and other predictors.	NA
Sample size	8	Explain how the study size was arrived at.	6,7
Missing data	9	Describe how missing data were handled (e.g., complete-case analysis, single imputation, multiple imputation) with details of any imputation method.	7
Statistical analysis methods	10a	Describe how predictors were handled in the analyses.	7
	10b	Specify type of model, all model-building procedures (including any predictor selection), and method for internal validation.	7,8
	10d	Specify all measures used to assess model performance and, if relevant, to compare multiple models.	7,8
Risk groups	11	Provide details on how risk groups were created, if done.	8
**Results**			
Participants	13a	Describe the flow of participants through the study, including the number of participants with and without the outcome and, if applicable, a summary of the follow-up time. A diagram may be helpful.	8
	13b	Describe the characteristics of the participants (basic demographics, clinical features, available predictors), including the number of participants with missing data for predictors and outcome.	8
Model development	14a	Specify the number of participants and outcome events in each analysis.	8
	14b	If done, report the unadjusted association between each candidate predictor and outcome.	8
Model specification	15a	Present the full prediction model to allow predictions for individuals (i.e., all regression coefficients, and model intercept or baseline survival at a given time point).	9
	15b	Explain how to the use the prediction model.	9
Model performance	16	Report performance measures (with CIs) for the prediction model.	9
**Discussion**			
Limitations	18	Discuss any limitations of the study (such as nonrepresentative sample, few events per predictor, missing data).	10
Interpretation	19b	Give an overall interpretation of the results, considering objectives, limitations, and results from similar studies, and other relevant evidence.	11
Implications	20	Discuss the potential clinical use of the model and implications for future research.	11,12
**Other information**			
Supplementary information	21	Provide information about the availability of supplementary resources, such as study protocol, Web calculator, and data sets.	13
Funding	22	Give the source of funding and the role of the funders for the present study.	13

We recommend using the TRIPOD Checklist in conjunction with the TRIPOD Explanation and Elaboration document.

BMJ Open

Developing a clinical prediction rule for repeated consultations with functional symptoms in primary care, a cohort study

Journal:	*BMJ Open*
Manuscript ID	bmjopen-2020-040730.R1
Article Type:	Original research
Date Submitted by the Author:	05-Aug-2020
Complete List of Authors:	Holtman, G.; UMCG, Department of general practice and elderly care medicine<br>Burger, H; UMCG<br>Verheij, Robert A; NIVEL, Netherlands Institute for Health Services Research<br>Wouters, Hans<br>Berger, Marjolein; UMCG, General Practice<br>Rosmalen, Judith; UMCG<br>Verhaak, Peter FM; UMCG
**Primary Subject Heading**:	General practice / Family practice
Secondary Subject Heading:	Epidemiology
Keywords:	EPIDEMIOLOGY, PRIMARY CARE, GENERAL MEDICINE (see Internal Medicine)

SCHOLARONE™
Manuscripts

I, the Submitting Author has the right to grant and does grant on behalf of all authors of the Work (as defined in the below author licence), an exclusive licence and/or a non-exclusive licence for contributions from authors who are: i) UK Crown employees; ii) where BMJ has agreed a CC-BY licence shall apply, and/or iii) in accordance with the terms applicable for US Federal Government officers or employees acting as part of their official duties; on a worldwide, perpetual, irrevocable, royalty-free basis to BMJ Publishing Group Ltd ("BMJ") its licensees and where the relevant Journal is co-owned by BMJ to the co-owners of the Journal, to publish the Work in this journal and any other BMJ products and to exploit all rights, as set out in our licence.

The Submitting Author accepts and understands that any supply made under these terms is made by BMJ to the Submitting Author unless you are acting as an employee on behalf of your employer or a postgraduate student of an affiliated institution which is paying any applicable article publishing charge ("APC") for Open Access articles. Where the Submitting Author wishes to make the Work available on an Open Access basis (and intends to pay the relevant APC), the terms of reuse of such Open Access shall be governed by a Creative Commons licence – details of these licences and which Creative Commons licence will apply to this Work are set out in our licence referred to above.

Other than as permitted in any relevant BMJ Author's Self Archiving Policies, I confirm this Work has not been accepted for publication elsewhere, is not being considered for publication elsewhere and does not duplicate material already published. I confirm all authors consent to publication of this Work and authorise the granting of this licence.

For peer review only

Developing a clinical prediction rule for repeated consultations with functional symptoms in primary care, a cohort study

G.A. Holtman[1], H. Burger[1], R.A. Verheij[2,3], H. Wouters[1,4], M.Y. Berger[1], J.G.M. Rosmalen[5], P.F.M. Verhaak[1,2]

[1] University of Groningen, University Medical Center Groningen, Department of General Practice and Elderly Care Medicine, Groningen, the Netherlands.

[2] Nivel, Netherlands Institute of Health Services Research, Utrecht, the Netherlands.

[3] Tilburg School of Social and Behavioral Sciences, Tranzo Scientific Center for Care and Welfare, Tilburg University, Tilburg, The Netherlands.

[4] General Practitioners Research Institute, Groningen, the Netherlands

[5] University of Groningen, University Medical Center Groningen, Interdisciplinary Center Psychopathology and Emotion Regulation, Groningen, the Netherlands.

Corresponding author: G.A. Holtman

Department of General Practice and Elderly Care Medicine,

University Medical Center Groningen,

University of Groningen,

PO Box 196, 9700 AD Groningen,

The Netherlands.

Telephone: 0031 642637125

E-mail address: g.a.holtman@umcg.nl

For peer review only

ABSTRACT

Objectives: Patients who present in primary care with chronic functional symptoms have reduced quality of life and increased health care costs. Recognizing these early is a challenge. The aim is to develop and internally validate a clinical prediction rule for repeated consultations with functional symptoms.

Design and setting: Records from the longitudinal population-based ("Lifelines") cohort study were linked to electronic health records from general practitioners (GPs).

Participants: We included patients consulting a GP with functional symptoms within one year after baseline assessment in the Lifelines cohort.

Outcome measures: The outcome is repeated consultations with functional symptoms, defined as ≥3 extra consultations for a functional symptom within 1 year after the first consultation. Multivariable logistic regression, with bootstrapping for internal validation, was used to develop a risk prediction model from 14 literature-based predictors. Model discrimination, calibration, and diagnostic accuracy were assessed.

Results: 18,810 participants were identified by database linkage, of whom 2,650 consulted a GP with functional symptoms and 297 (11%) had ≥3 extra consultations. In the final multivariable model, older age, female sex, lack of healthy activity, presence of generalized anxiety disorder, and higher number of GP consultations in the last year predicted repeated consultations. Discrimination after internal validation was 0.64 with a calibration slope of 0.95. The positive predictive value of patients with high scores on the model was 0.37 (0.29–0.47).

Conclusions: Several theoretically suggested predisposing and precipitating predictors, including neuroticism and stressful events, surprisingly failed to contribute to our final model. Moreover, this model mostly included general predictors of increased risk of repeated consultations among patients with functional symptoms. Moreover, the model discrimination and positive predictive values were insufficient and preclude clinical implementation.

Keywords: medically unexplained symptoms, cohort studies, clinical decision rules, primary health

For peer review only - http://bmjopen.bmj.com/site/about/guidelines.xhtml

care

Strengths and limitations of this study

- This study offers valuable insights into the predictors that could help general practitioners to identify repeated consultations with functional symptoms.
- By linking routine health care data from primary care to a large population-based cohort, we could include relevant predictors based on epidemiological and theoretical factors from the literature and this approach may serve to enhance primary care research in the future.
- Each patient had a full follow-up of 1 year.
- Time from baseline assessment of the population-based cohort to first GP consultation varied, however, taking this variance into account did not affect the magnitude of the coefficients of the predictors in a substantial way, nor their selection.
- We did not externally validate the model, however the performance need to be improved before such research can be considered.

For peer review only

INTRODUCTION

Functional symptoms represent those that cannot be explained by a physical disease and account for about a third of all presentations in primary care,[1,2] clustering as cardiopulmonary, musculoskeletal, gastrointestinal, and general somatic symptoms.[3,4] However, these clusters appear to correlate and considered to represent one condition with different manifestations.[5] Most patients with functional symptoms consult a general practitioner (GP) only once, but 10%–30% of cases will become chronic,[6] leading to more diagnostic tests, more referrals, higher health care costs, and more psychological distress compared with other patients.[7-9] Recognizing those patients at risk of developing chronic symptoms and consulted repeatedly the GP could therefore help to target interventions that reduce symptom severity,[10,11] improve quality of life, and reduce GP workloads. Ensuring that these patients are identified early is an important challenge facing GPs,[12] and one for which a validated clinical prediction rule may help. Several factors are known to increase the risk of chronicity of functional symptoms, including predisposing (e.g., neuroticism), precipitating (e.g., physical and psychosocial stressors), and perpetuating (e.g., lack of healthy physical activity) factors.[13-15] Despite being described in the literature,[6] these factors have yet to be combined in a clinical prediction rule for use in primary care.

In this study, we aimed to develop and internally validate a clinical prediction rule for repeated consultations among patients who consult GPs with functional symptoms.

METHOD

Data sources

We linked patient records from the Lifelines Cohort Study ("Lifelines")[16] with those from the Nivel Primary Care Database (NPCD).[17] Dutch law conditionally allows the use of such electronic health records for research purposes. Statistics Netherlands (CBS) then used temporary record identification numbers to link records at an individual level for analysis.

For peer review only - http://bmjopen.bmj.com/site/about/guidelines.xhtml

Lifelines is a multidisciplinary prospective population-based cohort study using a three-generation design to examine the health and health-related behaviors of 167,729 people living in the north of the Netherlands.[16] It employs a broad range of investigative procedures to assess key factors that contribute to health and disease in the general population, focusing on multimorbidity and complex genetics. Lifelines was approved by the medical ethics committee of the University Medical Centre Groningen (2007/152) and was conducted in accordance with the Declaration of Helsinki. All participants signed an informed consent form.

The NPCD contains routinely recorded clinical data from GP consultations with patients, and is considered representative of the Dutch population.[17] The Dutch healthcare system is such that all non-institutionalized members of the population are registered with a general practice, which in turn, serves as a gatekeeping system through which patients must pass to access specialist care via GP referral.[18] In total, 528 general practices participated in 2019, and this study was approved according the Nivel Governance Code (number NZR0317.033).

For the current study, we included the baseline data of 152,728 adults enrolled in Lifelines between November 2006 and June 2013, and we linked these with the electronic health records of GP consultations for patients aged ≥18 years who consulted one of the 65 general practices in the north of the Netherlands that participated in the NPCD.

Patient population

We planned to include adults with functional symptoms considered at risk of consulting the GP repeatedly, which we defined as those having a GP consultation for a functional symptom in the year after their baseline assessment for Lifelines. The presence of functional symptoms was assessed based on the International Classification of Primary Care (ICPC) codes that related to the symptoms that Robbins et al. described (see Supplementary Table 1).[19]

Outcomes

The primary outcome was repeated consultations with functional symptoms, defined as ≥3 extra GP

For peer review only - http://bmjopen.bmj.com/site/about/guidelines.xhtml

consultations for one of the defined functional symptoms (Supplementary Table 1) during a year of follow-up after first consulting a GP with that symptom.[19,20] Complete follow-up data were recorded for all GP consultations in electronic health records, and we permitted the functional symptoms to vary between consultations.

Candidate predictors

We selected 14 predictors based on literature review and expert opinion: age, sex, neuroticism, chronic stress, life events, self-rated health, physical activity, body mass index (BMI), living alone, education, major depressive disorder (MDD), generalized anxiety disorder (GAD), and psychiatric or GP consultations in the 12 months before first consulting with functional symptoms.[6,21] The data for these predictors were derived from the baseline of Lifelines, except for the psychiatric and GP consultations, which were derived from the NPCD.

Neuroticism was evaluated using an abridged version of the Neuroticism Extraversion Openness–Personality Inventory–Revised that included only anger-hostility, self-consciousness, impulsivity, and vulnerability, and excluded depression and anxiety (score range, 4–32).[22] Chronic stress was measured with the Long-term Difficulties Inventory (score range, 0–24).[23] The List of Threatening Events was used to assess the occurrence of 12 stressful life events (score range, 0–12).[23,24] Self-rated health was evaluated with the RAND-36 question[25] “how would you rate your health from 1 (excellent) to 5 (poor).” The Short Questionnaire to Assess Health-Enhancing Physical Activity was used to determine physical activity behavior, with a cut-off of 30 minutes at least 5 days a week indicating healthy activity.[26] Body weight and height were used to calculate BMI (weight (kg)/height (m^2)). Higher education was defined as at least secondary vocational education or work-based training. MDD and GAD were assessed by the Mini-International Neuropsychiatric Interview, compatible with International Classification of Disease, Tenth Edition, and the Diagnostic and Statistical Manual of Mental Disorders, Fourth Edition.[27] Psychiatric consultations was defined as patients with a consultation code in the P chapter of the ICPC and GP consultations was defined as

For peer review only

the number of total GP consultations in the 12 months before baseline of Lifelines.

Sample size

We estimated that we required 11,455 participants based on an assumption that 10% of the NPCD cohort would participate in Lifelines and that 75% of these data could be linked (i.e., 10% × 75% × 152,728). Given that the prevalence of repeated consultations with functional symptoms has been reported to be 2.5%, we estimated that 286 of these could be included[20] to achieve an effective sample size of at least 20 outcome events per predictor.[28]

Missing data

Eleven predictors from Lifelines had missing data, so we evaluated the underlying causes and patterns to assess the conditions for multiple imputation.[29] We checked predictors of missingness and we assumed missing at random (MAR) when patients with missing values were different from patients without missing values with respect to observed variables. When data is MAR, we replaced all missing values by multiple imputation by chained equations (MICE), incorporating all variables used in the analyses, including the outcome variable, and all variables that predicted missingness of a certain variable or value. We imputed questionnaire sum scores rather than item scores. Finally, we constructed 20 imputed datasets combined across all datasets, pooled β coefficients, and calculated odds ratios using Rubin's rule.[30]

Statistical analysis

Repeated consulations with functional symptoms over a one-year follow-up period was set as the binary outcome variable and associated with potential predictors as independent variables in logistic regression analyses. We performed univariable analyses to calculate unadjusted odds ratios.

To develop the clinical prediction rule, we initially included all potential predictors in a multivariable logistic regression model, irrespective of their univariable association and refrained from univariable preselection of candidate predictors to prevent model instability.[31] Using backward stepwise

For peer review only

selection, we excluded predictors from the model that were not statistically significant according to Akaike's information criterion (i.e., $p > 0.157$) in >50% of all imputed datasets.[32] Time in days between baseline assessment of predictors and first consultation differed between participants, so we also evaluated its influence in a separate analysis. We assessed rule performance by its discriminatory power with the C statistic and the calibration slope. We internally validated the model to correct for over-optimism by bootstrapping 250 samples, calculating a shrinkage factor, multiplying the original β coefficients by this factor, and re-estimating the intercepts using the shrunken β coefficients. The β coefficients were translated into a risk score of whole numbers for ease of use by GPs when evaluating the risk of repeated consultations in clinical practice. To that end, each β coefficient was divided by the coefficient closest to zero and then rounded to the nearest integer. The total score for each patient was calculated as the sum of all points for each predictor. We calculated the sensitivity, specificity, and positive predictive value of the rule at several thresholds to distinguish high and low risk. Thresholds were chosen arbitrarily based on the sample sizes being adequate in each category and the clinical risk being distinguishable.

All statistical analyses were performed with STATA/SE15 (STATA Corp, College station, TX, USA) and R (for bootstrapping). The Transparent Reporting of a multivariable prediction model for Individual Prediction of Diagnosis (TRIPOD) was used to conduct this study and report its results.[33]

Patient and public Involvement

Lifelines has a participant advisory board of eight active members with different background since 2016. The concept of this study was discussed during a meeting with this board. All Lifelines participants will receive the results of the study via a newsletter.

RESULTS

Study participants

For peer review only

For peer review only - http://bmjopen.bmj.com/site/about/guidelines.xhtml

Of the 152,728 Lifelines participants with a baseline assessment, we linked 18,810 (12%) with NPCD data (Figure 1). Among these, we included 2,650 participants (14% of those linked) attending GP consultations for a functional symptoms (i.e., the at-risk group), of whom 297 (11%) had ≥3 further consultations for functional symptoms (i.e., the outcome criterion). The details of the included and excluded patients are summarized in Table 1, showing that the groups were broadly comparable. Notably, 24% of participants had a missing value and 3% had missing values for >4 predictors. The participants with missing values were slightly older and less active, and they less often had completed higher education (Supplementary Table 2).

Clinical rule development

Univariable associations of the potential predictors for repeated consultations with functional symptoms are listed in Table 2. In the final multivariable model, the following five predictors were selected based on increasing the risk of repeated consultations: higher age, female sex, lack of healthy activity, presence of GAD, and having had more GP consultations in the year before first consulting with functional symptoms (Table 3). Adjustment for time from baseline to first consultation did not affect the magnitude of the coefficients of the predictors in a substantial way, nor their selection. The shrinkage factor of 0.95 showed limited model overfitting and was applied to adjust predictor coefficients in the final model. Likewise, the C statistic (area under the curve) of 0.65 (95% CI, 0.62–0.69) was corrected to 0.64 (95% CI, 0.61–0.68). Agreement between the observed and predicted proportion of events showed adequate calibration (Supplementary Figure 1).

The final model could calculate the absolute predicted individual risk of repeated consultations with functional symptoms (Supplemental Figure 2). For a risk score ≥100, the positive predictive value of repeated consultations was 0.37 (95% CI, 0.29–0.47) (Tables 4 and 5). However, when increasing the cut-off from 25 to 100, the sensitivity decreased from 0.87 (95% CI, 0.83–0.91) to 0.13 (95% CI, 0.10–0.17) and the specificity increased from 0.23 (95% CI, 0.22–0.25) to 0.97 (95% CI, 0.97–0.98).

For peer review only

DISCUSSION

Summary

We developed and internally validated a clinical prediction rule to identify patients at high risk of repeated consultations with functional symptoms. This was based on five factors that are readily available in primary care: age, sex, activity levels, GAD diagnosis, and number of consultations. However, despite being well calibrated, the prediction rule showed poor discrimination. Nevertheless, if patients scored ≥100, the risk of repeated consultations with functional symptoms increased to 37% from the baseline value of 11%.

Strengths and limitations

The study benefited from the use of a rich data set established by linking routine electronic health record data from primary care to a large population-based cohort. We effectively linked 18,810 patients (12%) from Lifelines who had at least one GP consultation, and we could include predictors based on epidemiological and theoretical factors from the literature, such as neuroticism and threatening events.[21] However, there are other relevant predictors that were not evaluated in Lifelines and were not requested from the NPCD data, such as panic disorder, lack of mastery, medically unexplained physical symptoms episodes, and psychiatric medication (tranquilizers and antidepressants).[34] The data linkage approach that we adopted may serve to enhance primary care research in the future. Each patient also had follow-up data for a full year, and although the time from baseline assessment in Lifelines to first GP consultation varied because of the dynamic nature of the NPCD cohort, this did not affect the results. Another strength is that we included 21 events per variable, resulting in minimal overfitting with a shrinkage factor of 0.95. An advantage of using dichotomous over continuous outcomes is that clinical interpretation is more straightforward. Although it is problematic that we did not externally validate the model, we contend that the model's performance will need to be improved before such research can be considered.

For peer review only - http://bmjopen.bmj.com/site/about/guidelines.xhtml

Our model predicts the risk of having ≥3 extra consultations for functional symptoms. However, this should not be confused with predicting a somatic symptom disorder or functional somatic syndrome, not least because we could not determine these psychiatric diagnoses with the available data. In addition, about 80% of patients with functional somatic syndrome will be missed using GP medical files.[35] A disadvantage of our outcome measure is that patients with functional symptoms may also have consulted other health care professionals (e.g., physiotherapist), so these cases may have been missed. Therefore, the interpretation of our model is only applicable for GP consultations. We chose to use a follow-up of 1 year as this is often used in previous studies,[20,36] however persistent frequent attenders in primary care have more often functional somatic symptoms.[37] Therefore, a clinical prediction rule for repeated consultations with functional symptoms during a longer follow-up might perform better. To avoid confusing and misunderstanding, we used the more neutral outcome of repeated consultations because our data did not allow for the identification of frequent attenders as defined in present day literature. The latter requires a comprehensive description of the contacts counted, e.g. how many were out-of-hours contacts, and how many were administrative or preventive consultations.[36] Our approach to identify the at-risk population first may explain the contrast with existing data. For example, we showed that 11% of patients presenting with functional symptoms ultimately had ≥4 consultations for these symptoms, whereas previous research has shown a rate of 2.5% among all patients with GP consultations.[20]

Comparison with other studies

We are aware of no other clinical prediction rules for repeated GP consultations with functional symptoms. It should be emphasized that such a model cannot be considered synonymous with explaining the cause.[38] We found three studies that developed models by combining predictors using a backward or forward selection procedure.

One study used information from GP letters to medical specialists for patients who were referred with functional somatic symptoms.[39] In their clinical prediction rule, female sex, referral symptom

For peer review only - http://bmjopen.bmj.com/site/about/guidelines.xhtml

group, lack of somatic comorbidity, lack of abnormal physical findings, history of psychiatric diagnosis or treatment, and referral letter written in illness terminology were all shown to be predictors. This model had a higher area under the curve (0.80) than ours (0.64) and was developed for patients consulting internists. However, the GP referral letters included relevant predictors that helped to identify functional somatic symptoms, and although the population was more selected than ours, the results show that data collected in primary care can be suitable predictors.

Another study that showed that routine health care could include relevant predictors, developed a clinical prediction rule that potentially could be used to identify patients at risk for persistent functional somatic symptoms from routine primary care medical records.[40] The model had an area under the curve of 0.70 and the most important discriminative variable for persistent functional somatic symptoms was number of episodes. Just like our model they also included the predictors age, sex, and number of contacts. Other selected predictors were physiotherapy, number of referrals, some medications, number of prescription products with regards to medication, number of trade products with regards to medication, free text questions, and number of laboratory results.

The other study developed a model for symptom severity and for both physical and mental functioning during a 2-year follow-up period among patients with functional symptoms.[21] They predicted severe courses by physical comorbidity, higher baseline severity and longer functional symptom duration, anxiety, catastrophizing cognitions, embarrassment, and neuroticism, as well as fear avoidance, avoidance, or resting behavior. By contrast, they predicted favorable courses based on limited alcohol use, higher education, higher baseline physical and mental functioning, symptom focusing, damage cognitions, and extraversion. Although we also identified anxiety as a predictor, we did not find the same for neuroticism or higher education. Also contrasting with our data, as well as that of others,[41,42] they did not show that female sex was a predictor. Unfortunately, we could not include predictors of illness cognitions or attitude because these were not evaluated in Lifelines. Indeed, the Symptoms Checklist 90 questionnaire had more than 50% missing values during baseline evaluation in Lifelines, so we excluded these data.[16] The differences in identified predictors may be

For peer review only - http://bmjopen.bmj.com/site/about/guidelines.xhtml
For peer review only

explained by different study populations, predictor selection criteria, or outcomes.

Implications for research and practice

To our surprise, several theoretically suggested predisposing and precipitating predictors, including neuroticism and stressful events, failed to contribute to the final prediction model. Instead, this model included mostly general predictors that provide little additional information to help GPs recognize patients at risk of consulting repeatedly with functional symptoms, and it not only has poor discrimination and positive predictive value but also lacks external validation. Therefore, at present, we cannot recommend the score for clinical use. Nevertheless, our findings indicate that GPs might expect chronicity when older women with low activity levels and anxiety symptoms present with functional symptoms. These require extra vigilance and may benefit from early intervention with self-help advice.[11] Some predictors identified in earlier studies, such as female sex and anxiety, could be potential factors in future clinical prediction rules designed to help GPs recognize patients at risk of consulting the GP repeatedly.

For peer review only - http://bmjopen.bmj.com/site/about/guidelines.xhtml

FUNDING

This study was funded by a grant from the Healthy Ageing Pilot of the University Medical Center Groningen (CDO17.0002/2017-1/296). The Lifelines initiative has been made possible by support from the Dutch Ministry of Health, Welfare and Sport; the Dutch Ministry of Economic Affairs; University Medical Center Groningen; Groningen University; and the Provinces in the north of the Netherlands (Drenthe, Friesland, Groningen).

ETHICAL APPROVAL

The medical ethics committee of the University Medical Centre Groningen approved the Lifelines study (2007/152). Dutch law conditionally allows the use of electronic health records for research purposes.

COMPETING INTERESTS

None declared.

PATIENT CONSENT FORM

Not required.

AUTHOR CONTRIBUTORS:

Study concept and design: GAH, HB, PFMV. Acquisition of data: PFMV and RAV. Analysis of data: GAH. Interpretation of data: GAH, HB, RAV, HW, MYB, JGMR, PFMV. Drafting the work: GAH, HB, PFMV. The final manuscript was critically revised and approved by all authors.

DATA AVAILABILITY STATEMENT:

The data used is part of two ongoing databases: Lifelines Cohort Study and Nivel Primary Care Database. The data is not available. The corresponding author can be contacted for details.

WORD COUNT

2999 words

For peer review only

ACKNOWLEDGMENT

We thank Dr Robert Sykes (www.doctored.org.uk) for providing editorial services.

For peer review only

REFERENCES

1. Jackson JL, Passamonti M. The outcomes among patients presenting in primary care with a physical symptom at 5 years. *Journal of general internal medicine*. 2005;20(11):1032-1037.

2. Rosendal M, Carlsen AH, Rask MT, Moth G. Symptoms as the main problem in primary care: A cross-sectional study of frequency and characteristics. *Scand J Prim Health Care*. 2015;33(2):91-99.

3. Fink P, Toft T, Hansen MS, Ornbol E, Olesen F. Symptoms and syndromes of bodily distress: An exploratory study of 978 internal medical, neurological, and primary care patients. *Psychosom Med*. 2007;69(1):30-39.

4. Rosmalen J, Tak L, De Jonge P. Empirical foundations for the diagnosis of somatization: Implications for DSM-5. *Psychol Med*. 2011;41(6):1133-1142.

5. Henningsen P, Zipfel S, Sattel H, Creed F. Management of functional somatic syndromes and bodily distress. *Psychother Psychosom*. 2018;87(1):12-31.

6. olde Hartman TC, Borghuis MS, Lucassen PL, van de Laar, Floris A, Speckens AE, van Weel C. Medically unexplained symptoms, somatisation disorder and hypochondriasis: Course and prognosis. A systematic review. *J Psychosom Res*. 2009;66(5):363-377.

7. Dirkzwager AJ, Verhaak PF. Patients with persistent medically unexplained symptoms in general practice: Characteristics and quality of care. *BMC Family Practice*. 2007;8(1):33.

8. Burton C, McGorm K, Richardson G, Weller D, Sharpe M. Healthcare costs incurred by patients repeatedly referred to secondary medical care with medically unexplained symptoms: A cost of illness study. *J Psychosom Res*. 2012;72(3):242-247.

For peer review only

For peer review only - http://bmjopen.bmj.com/site/about/guidelines.xhtml

9. De Waal, Margot W M, Arnold IA, Eekhof JA, Van Hemert AM. Somatoform disorders in general practice: Prevalence, functional impairment and comorbidity with anxiety and depressive disorders. *The British Journal of Psychiatry*. 2004;184(6):470-476.

10. Van Dessel N, Den Boeft M, van der Wouden, Johannes C, et al. Nonâ€pharmacological interventions for somatoform disorders and medically unexplained physical symptoms (MUPS) in adults. *Cochrane Database of Systematic Reviews*. 2014(11).

11. van Gils A, Schoevers RA, Bonvanie IJ, Gelauff JM, Roest AM, Rosmalen JG. Self-help for medically unexplained symptoms: A systematic review and meta-analysis. *Psychosom Med*. 2016;78(6):728-739.

12. Rosendal M, Hartman TCO, Aamland A, et al. â€œMedically unexplainedâ€ symptoms and symptom disorders in primary care: Prognosis-based recognition and classification. *BMC family practice*. 2017;18(1):18.

13. Frølund Pedersen H, Frostholm L, Søndergaard Jensen J, Ørnbøl E, Schröder A. Neuroticism and maladaptive coping in patients with functional somatic syndromes. *British journal of health psychology*. 2016;21(4):917-936.

14. Salit IE. Precipitating factors for the chronic fatigue syndrome. *J Psychiatr Res*. 1997;31(1):59-65.

15. Van Houdenhove B, Luyten P. Customizing treatment of chronic fatigue syndrome and fibromyalgia: The role of perpetuating factors. *Psychosomatics*. 2008;49(6):470-477.

16. Scholtens S, Smidt N, Swertz MA, et al. Cohort profile: LifeLines, a three-generation cohort study and biobank. *Int J Epidemiol*. 2014;44(4):1172-1180.

17. NIVEL. Https://Www.nivel.nl/nl/nivel-zorgregistraties-eerste-lijn/nivel-zorgregistraties-eerste-lijn. https://www.nivel.nl/nl/nivel-zorgregistraties-eerste-lijn/nivel-zorgregistraties-eerste-lijn.

For peer review only - http://bmjopen.bmj.com/site/about/guidelines.xhtml

18. Linden M, Gothe H, Ormel J. Pathways to care and psychological problems of general practice patients in a “gate keeper” and an” open access” health care system. *Soc Psychiatry Psychiatr Epidemiol*. 2003;38(12):690-697.

19. Robbins JM, Kirmayer LJ, Hemami S. Latent variable models of functional somatic distress. *J Nerv Ment Dis*. 1997;185(10):606-615.

20. Verhaak PF, Meijer SA, Visser AP, Wolters G. Persistent presentation of medically unexplained symptoms in general practice. *Fam Pract*. 2006;23(4):414-420.

21. Claassen-van Dessel N, van der Wouden, Johannes C, Twisk JW, Dekker J, van der Horst, Henriëtte E. Predicting the course of persistent physical symptoms: Development and internal validation of prediction models for symptom severity and functional status during 2 years of follow-up. *J Psychosom Res*. 2018;108:1-13.

22. Costa PT, Mac Crae RR. *Neo personality inventory-revised (NEO PI-R).* Psychological Assessment Resources Odessa, FL; 1992.

23. Rosmalen J, Bos E, De Jonge P. Validation of the long-term difficulties inventory (LDI) and the list of threatening experiences (LTE) as measures of stress in epidemiological population-based cohort studies. *Psychol Med*. 2012;42(12):2599-2608.

24. Brugha TS, Cragg D. The list of threatening experiences: The reliability and validity of a brief life events questionnaire. *Acta Psychiatr Scand*. 1990;82(1):77-81.

25. Hays RD, Morales LS. The RAND-36 measure of health-related quality of life. *Ann Med*. 2001;33(5):350-357.

For peer review only - http://bmjopen.bmj.com/site/about/guidelines.xhtml

26. Wendel-Vos GW, Schuit AJ, Saris WH, Kromhout D. Reproducibility and relative validity of the short questionnaire to assess health-enhancing physical activity. *J Clin Epidemiol*. 2003;56(12):1163-1169.

27. Sheehan DV, Lecrubier Y, Sheehan KH, et al. The mini-international neuropsychiatric interview (MINI): The development and validation of a structured diagnostic psychiatric interview for DSM-IV and ICD-10. *J Clin Psychiatry*. 1998.

28. Peduzzi P, Concato J, Kemper E, Holford TR, Feinstein AR. A simulation study of the number of events per variable in logistic regression analysis. *J Clin Epidemiol*. 1996;49(12):1373-1379.

29. Hughes RA, Heron J, Sterne JA, Tilling K. Accounting for missing data in statistical analyses: Multiple imputation is not always the answer. *Int J Epidemiol*. 2019;1:11.

30. Marshall A, Altman DG, Holder RL, Royston P. Combining estimates of interest in prognostic modelling studies after multiple imputation: Current practice and guidelines. *BMC Med Res Methodol*. 2009;9:57-2288-9-57.

31. Harrell Jr FE, Lee KL, Mark DB. Multivariable prognostic models: Issues in developing models, evaluating assumptions and adequacy, and measuring and reducing errors. *Stat Med*. 1996;15(4):361-387.

32. Sauerbrei W. The use of resampling methods to simplify regression models in medical statistics. *Journal of the Royal Statistical Society: Series C (Applied Statistics)*. 1999;48(3):313-329.

33. Collins GS, Reitsma JB, Altman DG, Moons KG. Transparent reporting of a multivariable prediction model for individual prognosis or diagnosis (TRIPOD): The TRIPOD statement. *BMC medicine*. 2015;13(1):1.

For peer review only

34. Smits FT, Brouwer HJ, Zwinderman AH, et al. Why do they keep coming back? psychosocial etiology of persistence of frequent attendance in primary care: A prospective cohort study. *J Psychosom Res*. 2014;77(6):492-503.

35. den Boeft M, van der Wouden, Johannes C, Rydell-Lexmond TR, de Wit NJ, van der Horst, Henriëtte E, Numans ME. Identifying patients with medically unexplained physical symptoms in electronic medical records in primary care: A validation study. *BMC family practice*. 2014;15(1):109.

36. Vedsted P, Christensen MB. Frequent attenders in general practice care: A literature review with special reference to methodological considerations. *Public Health*. 2005;119(2):118-137.

37. Smits FT, Brouwer HJ, ter Riet G, van Weert HC. Epidemiology of frequent attenders: A 3-year historic cohort study comparing attendance, morbidity and prescriptions of one-year and persistent frequent attenders. *BMC Public Health*. 2009;9(1):36.

38. Moons KG, Royston P, Vergouwe Y, Grobbee DE, Altman DG. Prognosis and prognostic research: What, why, and how? *BMJ*. 2009;338:b375.

39. Gol JM, Burger H, Janssens KA, Slaets JP, Gans RO, Rosmalen JG. PROFSS: A screening tool for early identification of functional somatic symptoms. *J Psychosom Res*. 2014;77(6):504-509.

40. den Boeft M. Medically unexplained physical symptoms in primary care: Identification, management and societal aspects. 2016.

41. Speckens A, Van Hemert A, Bolk J, Rooijmans H, Hengeveld M. Unexplained physical symptoms: Outcome, utilization of medical care and associated factors. *Psychol Med*. 1996;26(4):745-752.

42. Leiknes KA, Finset A, Moum T, Sandanger I. Methodological issues concerning lifetime medically unexplained and medically explained symptoms of the composite international diagnostic interview: A prospective 11-year follow-up study. *J Psychosom Res*. 2006;61(2):169-179.

For peer review only - http://bmjopen.bmj.com/site/about/guidelines.xhtml

For peer review only

For peer review only

Figure 1. Patient selection flowchart

Note: extra consultations (≥3) refers to additional presentations for functional symptoms during a one-year follow-up period after an initial GP consultation for a functional symptom.

For peer review only

Table 1. Characteristics of included and excluded patients

	Included patients[a] (n = 2,650)		Excluded patients[b] (n = 7,418)	
	N (%)	Score	*n*	Score
Age, mean y (SD)	2,636 (99)	45 (14)	7,387	45 (14)
Female, n (%)	2,650 (100)	1,802 (68)	7,418	4,577 (62)
Neuroticism, median (IQR)	2,248 (85)	10.1 (9.1-11.3)	6,419	9.9 (8.9 – 11)
Chronic stress, median (IQR)	2,465 (93)	2 (1–4)	7,005	2 (1–4)
Threatening events, median (IQR)	2,464 (93)	1 (0–2)	7,008	1 (0–2)
Self-rated health, median (IQR)	2,548 (96)	3 (2–3)	7,228	3 (2–3)
Healthy activity[c], n (%)	2,274 (86)	1,259 (55)	6,505	3,589 (55)
Body mass index (kg/m^2), median (IQR)	2,648 (100)	26 (23–28)	7,417	25 (23–28)
Living alone, n (%)	2,522 (95)	335 (13.3)	7,167	904 (12.6)
Higher education[d], n (%)	2,572 (97)	1,707 (66)	7,215	5,067 (70)
MDD[f], n (%)	2,555 (96)	86 (3)	7,248	176 (2.4)
GAD[g], n (%)	2,555 (96)	165 (6)	7,248	351 (4.8)
Psychiatric consultations last year[h,i], n (%)	2,650 (100)	292 (11)	7,418	783 (11)
GP consultations last year[i], median (IQR)	2,650 (100)	2 (0–5)	7,418	1 (0–3)

[a] Included: GP consultations and ≥1 functional symptom within 1 year after baseline Lifelines assessment.
[b] Excluded: GP consultation without a functional symptom within 1 year after baseline of Lifelines.
[c] Healthy activity, defined as 30 minutes at least 5 days a week.
[d] Higher education, defined as at least secondary vocational education or work-based training.
[f] MDD: Major Depressive Disorder.
[g] GAD: Generalized Anxiety Disorder.
[h] Patients with a consultation code in the P chapter of the International Classification of Primary Care.
[i] Predictors from NPCD. Other predictors are from Lifelines.
Abbreviations: SD, standard deviation; IQR, Interquartile Range; GP, general practitioner

For peer review only - http://bmjopen.bmj.com/site/about/guidelines.xhtml

Table 2. Univariable analysis of predictors for repeated consultations with functional symptoms

Variable	Repeated consultations[a] OR (95% CI)
Age	1.01 (1.00–1.02)
Sex (m)	0.69 (0.52–0.91)
Neuroticism	1.08 (0.99–1.17)
Chronic stress	1.04 (0.99–1.09)
Threatening events	1.08 (0.99–1.18)
Self-rated health	1.41 (1.20–1.66)
Healthy activity[b]	0.70 (0.53–0.91)
Body mass index (kg/m^2)	1.04 (1.01–1.07)
Living alone	0.91 (0.62–1.32)
Higher education[c]	0.75 (0.58–0.96)
MDD[d]	1.53 (0.85–2.73)
GAD[e]	2.15 (1.45–3.19)
Psychiatric consultations last year[f,g]	1.17 (1.04–1.33)
GP consultations last year[g]	1.12 (1.09–1.15)

[a] Outcome, ≥3 extra functional symptom consultations during a 1-year follow-up period (n = 297).
[b] Healthy activity, defined as 30 minutes at least 5 days a week.
[c] Higher education, defined as at least secondary vocational education or work-based training.
[d] MDD: Major Depressive Disorder.
[e] GAD: Generalized Anxiety Disorder.
[f] Number of consultations concerning International Classification of Primary Care codes in the P chapter.
[g] Predictors are from NPCD and are continuous. Other predictors are from the Lifelines database.
Abbreviations: CI, confidence interval; GP, general practitioner

For peer review only

Table 3. Final multivariable analysis for repeated consultations with functional symptoms

Predictors	OR (95% CI) [c]	P-value	Coefficient	Adjusted coefficient	Risk score
Constant	0.05 (0.03–0.08)	0.000	-2.95	-3.80	
Age	1.02 (1.01–1.03)	0.000	0.02	0.02	1
Sex (m)	0.75 (0.56–0.99)	0.042	-0.30	-0.29	-15
Healthy activity[a]	0.60 (0.45–0.80)	0.001	-0.51	-0.48	-24
GAD[b]	1.79 (1.17–2.74)	0.008	0.58	0.56	28
GP consultations last year	1.10 (1.07–1.14)	0.000	0.10	0.10	5

[a] Healthy activity, defined as 30 minutes at least 5 days a week.
[b] GAD: Generalized Anxiety Disorder.
[c] OR (95% CI): Odds Ratio (95% confidence interval).
Note: Shrinkage factor 0.96; predictors selected if P < 0.157.
Abbreviations: CI, confidence interval; GP, general practitioner

For peer review only

Table 4. Risk of repeated consultations with functional symptoms by different cut-off scores

Cut-off score	*n*	Outcome	Observed risk	Predicted risk
<25	585	38	0.07	0.06
25–49	1009	82	0.08	0.08
50–99	952	138	0.15	0.14
≥100	104	39	0.38	0.37

Note: the risk score was calculated by multiplying each risk score by the predictor value, with the total score ranging from −21 to 301 for all included patients (for example -21 represents the following patient: 18 years (18), male (-15), healthy activity (-24), no GAD (0), no GP consultation last year (0) (=18-15-24+0+0=-21) and 301: 63 years (63), female (0), lack of healthy activity (0), presence of GAD (28), 42 GP consultations last year (210) (=63+0+0+28+210=301)).

For peer review only

Table 5. Diagnostic accuracy of the risk score for repeated consultations with functional symptoms

Cut-off score	*n*	Sensitivity (95% CI) [c]	Specificity (95% CI) [c]	PPV (95% CI)	NPV (95% CI)
≥25	2065	0.87 (0.83–0.91)	0.23 (0.22–0.25)	0.13 (0.11–0.14)	0.93 (0.91–0.95)
≥50	1057	0.59 (0.54–0.65)	0.63 (0.61–0.64)	0.17 (0.15–0.19)	0.92 (0.91–0.94)
≥100	104	0.13 (0.10–0.17)	0.97 (0.97–0.98)	0.37 (0.29–0.47)	0.91 (0.90–0.92)

Abbreviations: PPV, positive predictive value; NPV, negative predictive value, CI, confidence interval.

For peer review only

For peer review only - http://bmjopen.bmj.com/site/about/guidelines.xhtml

For peer review only

For peer review only - http://bmjopen.bmj.com/site/about/guidelines.xhtml

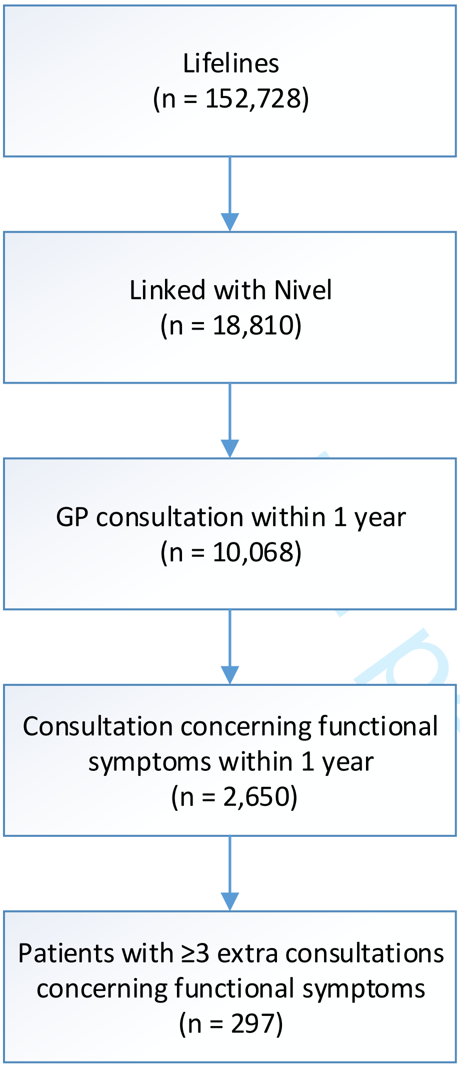

For peer review only

Supplementary Table 1. Functional symptoms and the corresponding International Classification of Primary Care codes

Symptom	International Classification of Primary Care code
Generalized pain	A01
Fatigue/Tiredness/weakness	A04
Abdominal pain	D01
Flatulence	D08
Nauseous	D09
Constipation	D12
Defecation problems	D18
Irritable Bowel Syndrome	D93
Chest pain	K01; K02
Palpitations	K04
Joint pain	L01; L08
Back pain	L02; L03
Extremities pain	L09; L14
Headache	N01; N02
Wheezy	N02
Dizzy	N20
Sleep disorder	P06
Concentration problems	P20
Sore Throat	R21
Loss in appetite	T03
Weight gain/loss	T07; T08

For peer review only

For peer review only - http://bmjopen.bmj.com/site/about/guidelines.xhtml

Supplementary Table 2. Comparison of patients with and without missing values

	No missing values (n = 2,002)		Missing values (n = 648)		Univariable OR (95% CI)
	n	value	*n*	value	
Age, mean y (SD)	2,002	42 (12)	634	54 (17)	**1.07 (1.06–1.07)**
Male, n (%)	2,002	622 (31)	648	226 (35)	1.19 (0.99–1.43)
Neuroticism, median (IQR)	2,002	10.1 (9.1–11.3)	246	10.3 (9.3–11.5)	1.04 (0.96–1.12)
Chronic stress, median (IQR)	2,002	3 (1–4)	463	2 (0–4)	**0.90 (0.86–0.94)**
Threatening events, median (IQR)	2,002	1 (0–2)	462	1 (0–2)	**1.09 (1.02–1.17)**
Self-rated health, median (IQR)	2,002	3 (2–3)	546	3 (2–3)	1.10 (0.97–1.25)
Healthy activity[a], n (%)	2,002	1,056 (53)	272	203 (75)	**2.63 (1.98–3.51)**
Body mass index (kg/m^2), median (IQR)	2,002	25 (23–28)	646	26 (24–29)	**1.05 (1.03–1.07)**
Living alone, n (%)	2,002	233 (12)	520	102 (20)	**1.85 (1.43–2.39)**
Higher education[b], n (%)	2,002	1,412 (71)	570	295 (52)	**0.45 (0.37–0.54)**
MDD[c], n (%)	2,002	64 (3)	553	22 (4)	1.25 (0.77–2.06)
GAD[d], n (%)	2,002	138 (7)	553	27 (5)	0.69 (0.45–1.06)
Psychiatric consultations last year[e,f], n (%)	2,002	205 (10)	648	87 (13)	1.07 (0.96–1.19)
GP consultations last year[f], median (IQR)	2,002	2 (0–5)	648	3 (1–6)	**1.05 (1.03–1.08)**
Repeated consultations[g], n (%)	2,002	200 (10)	648	97 (15)	**1.59 (1.22–2.06)**

[a] Healthy activity, defined as 30 minutes at least 5 days a week.
[b] Higher education, defined as at least secondary vocational education or work-based training.
[c] MDD: Major Depressive Disorder.
[d] GAD: Generalized Anxiety Disorder.
[e] Patients with a consultation code in the P chapter of the International Classification of Primary Care.
[f] Predictors from NPCD and both are continuous variables. Other predictors are from Lifelines database.
[g] Repeated consultations is defined as ≥3 extra functional symptom consultations during one year of follow-up.
Note: 24% (648/2,650) had a missing value
Abbreviations: SD, standard deviation; IQR, Interquartile Range; GP, general practitioner; OR, odds ratio

For peer review only

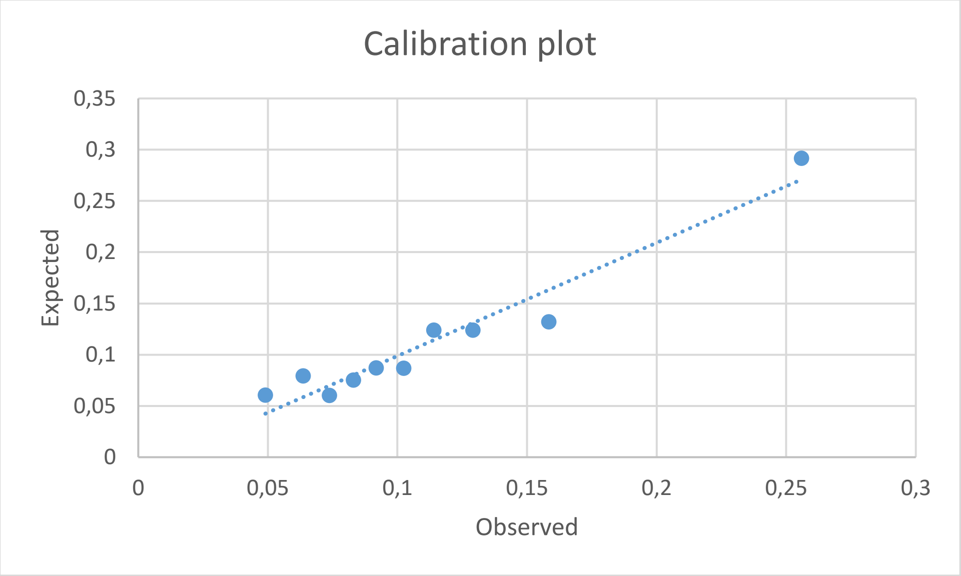

Supplementary Figure 1. Calibration plot of the clinical prediction rule for repeated consultations with functional symptoms

For peer review only

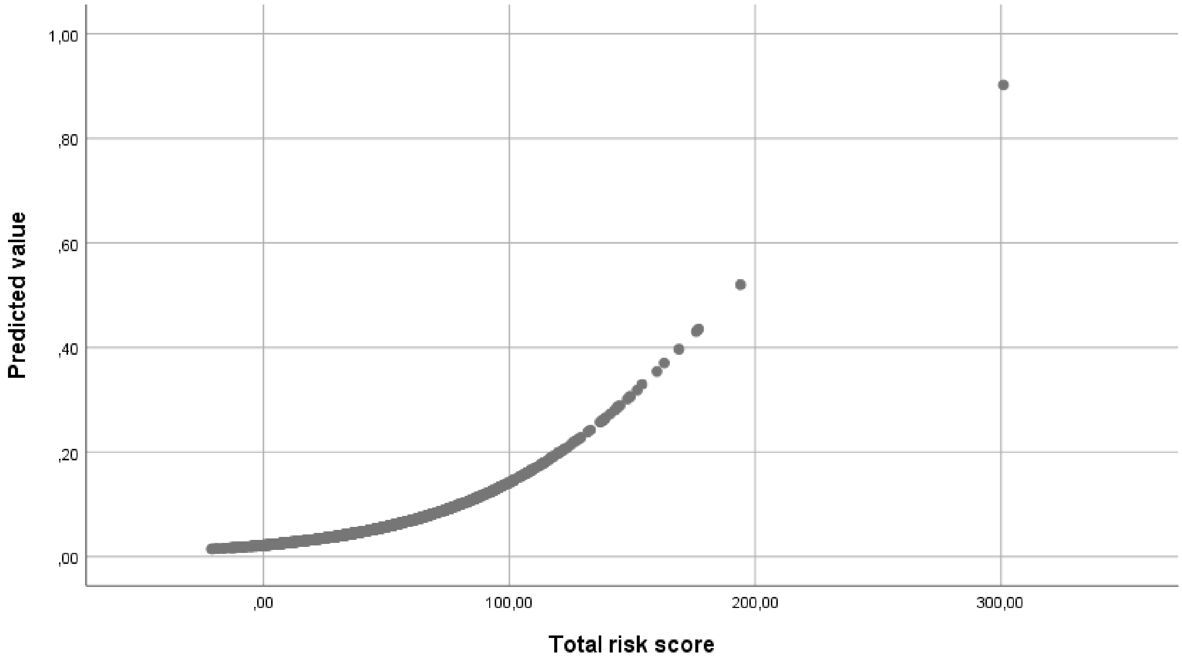

Supplemental Figure 2. Relation between the total risk score and the predicted risk of repeated consultations with functional symptoms

For peer review only

TRIPOD Checklist: Prediction Model Development

Section/Topic	Item	Checklist Item	Page
**Title and abstract**			
Title	1	Identify the study as developing and/or validating a multivariable prediction model, the target population, and the outcome to be predicted.	1
Abstract	2	Provide a summary of objectives, study design, setting, participants, sample size, predictors, outcome, statistical analysis, results, and conclusions.	2
**Introduction**			
Background and objectives	3a	Explain the medical context (including whether diagnostic or prognostic) and rationale for developing or validating the multivariable prediction model, including references to existing models.	3
	3b	Specify the objectives, including whether the study describes the development or validation of the model or both.	3
**Methods**			
Source of data	4a	Describe the study design or source of data (e.g., randomized trial, cohort, or registry data), separately for the development and validation data sets, if applicable.	4,5
	4b	Specify the key study dates, including start of accrual; end of accrual; and, if applicable, end of follow-up.	5,6
Participants	5a	Specify key elements of the study setting (e.g., primary care, secondary care, general population) including number and location of centres.	5
	5b	Describe eligibility criteria for participants.	5
	5c	Give details of treatments received, if relevant.	NA
Outcome	6a	Clearly define the outcome that is predicted by the prediction model, including how and when assessed.	5,6
	6b	Report any actions to blind assessment of the outcome to be predicted.	NA
Predictors	7a	Clearly define all predictors used in developing or validating the multivariable prediction model, including how and when they were measured.	6
	7b	Report any actions to blind assessment of predictors for the outcome and other predictors.	NA
Sample size	8	Explain how the study size was arrived at.	6,7
Missing data	9	Describe how missing data were handled (e.g., complete-case analysis, single imputation, multiple imputation) with details of any imputation method.	7
Statistical analysis methods	10a	Describe how predictors were handled in the analyses.	7
	10b	Specify type of model, all model-building procedures (including any predictor selection), and method for internal validation.	7,8
	10d	Specify all measures used to assess model performance and, if relevant, to compare multiple models.	7,8
Risk groups	11	Provide details on how risk groups were created, if done.	8
**Results**			
Participants	13a	Describe the flow of participants through the study, including the number of participants with and without the outcome and, if applicable, a summary of the follow-up time. A diagram may be helpful.	8
	13b	Describe the characteristics of the participants (basic demographics, clinical features, available predictors), including the number of participants with missing data for predictors and outcome.	8
Model development	14a	Specify the number of participants and outcome events in each analysis.	8
	14b	If done, report the unadjusted association between each candidate predictor and outcome.	8
Model specification	15a	Present the full prediction model to allow predictions for individuals (i.e., all regression coefficients, and model intercept or baseline survival at a given time point).	9
	15b	Explain how to the use the prediction model.	9
Model performance	16	Report performance measures (with CIs) for the prediction model.	9
**Discussion**			
Limitations	18	Discuss any limitations of the study (such as nonrepresentative sample, few events per predictor, missing data).	10
Interpretation	19b	Give an overall interpretation of the results, considering objectives, limitations, and results from similar studies, and other relevant evidence.	11
Implications	20	Discuss the potential clinical use of the model and implications for future research.	11,12
**Other information**			
Supplementary information	21	Provide information about the availability of supplementary resources, such as study protocol, Web calculator, and data sets.	13
Funding	22	Give the source of funding and the role of the funders for the present study.	13

We recommend using the TRIPOD Checklist in conjunction with the TRIPOD Explanation and Elaboration document.

For peer review only - http://bmjopen.bmj.com/site/about/guidelines.xhtml

BMJ Open

Developing a clinical prediction rule for repeated consultations with functional somatic symptoms in primary care, a cohort study

Journal:	*BMJ Open*
Manuscript ID	bmjopen-2020-040730.R2
Article Type:	Original research
Date Submitted by the Author:	19-Nov-2020

Complete List of Authors:	Holtman, G.; UMCG, Department of general practice and elderly care medicine<br>Burger, H; UMCG, Department of General Practice and Elderly Care Medicine<br>Verheij, Robert A; NIVEL<br>Wouters, Hans; UMCG, Department of General Practice and Elderly Care Medicine; General Practitioners Research Institute<br>Berger, Marjolein; UMCG, Department of General Practice and Elderly Care Medicine<br>Rosmalen, Judith; UMCG, Interdisciplinary Center Psychopathology and Emotion Regulation<br>Verhaak, Peter FM; UMCG, Department of General Practice and Elderly Care Medicine; NIVEL

**Primary Subject Heading**:	General practice / Family practice
Secondary Subject Heading:	Epidemiology
Keywords:	EPIDEMIOLOGY, PRIMARY CARE, GENERAL MEDICINE (see Internal Medicine)

SCHOLARONE™
Manuscripts

I, the Submitting Author has the right to grant and does grant on behalf of all authors of the Work (as defined in the below author licence), an exclusive licence and/or a non-exclusive licence for contributions from authors who are: i) UK Crown employees; ii) where BMJ has agreed a CC-BY licence shall apply, and/or iii) in accordance with the terms applicable for US Federal Government officers or employees acting as part of their official duties; on a worldwide, perpetual, irrevocable, royalty-free basis to BMJ Publishing Group Ltd ("BMJ") its licensees and where the relevant Journal is co-owned by BMJ to the co-owners of the Journal, to publish the Work in this journal and any other BMJ products and to exploit all rights, as set out in our licence.

The Submitting Author accepts and understands that any supply made under these terms is made by BMJ to the Submitting Author unless you are acting as an employee on behalf of your employer or a postgraduate student of an affiliated institution which is paying any applicable article publishing charge ("APC") for Open Access articles. Where the Submitting Author wishes to make the Work available on an Open Access basis (and intends to pay the relevant APC), the terms of reuse of such Open Access shall be governed by a Creative Commons licence – details of these licences and which Creative Commons licence will apply to this Work are set out in our licence referred to above.

Other than as permitted in any relevant BMJ Author's Self Archiving Policies, I confirm this Work has not been accepted for publication elsewhere, is not being considered for publication elsewhere and does not duplicate material already published. I confirm all authors consent to publication of this Work and authorise the granting of this licence.

For peer review only

Developing a clinical prediction rule for repeated consultations with functional somatic symptoms in primary care, a cohort study

G.A. Holtman[1], H. Burger[1], R.A. Verheij[2,3], H. Wouters[1,4], M.Y. Berger[1], J.G.M. Rosmalen[5], P.F.M. Verhaak[1,2]

[1] University of Groningen, University Medical Center Groningen, Department of General Practice and Elderly Care Medicine, Groningen, the Netherlands.

[2] Nivel, Netherlands Institute of Health Services Research, Utrecht, the Netherlands.

[3] Tilburg School of Social and Behavioral Sciences, Tranzo Scientific Center for Care and Welfare, Tilburg University, Tilburg, The Netherlands.

[4] General Practitioners Research Institute, Groningen, the Netherlands

[5] University of Groningen, University Medical Center Groningen, Interdisciplinary Center Psychopathology and Emotion Regulation, Groningen, the Netherlands.

Corresponding author: G.A. Holtman

Department of General Practice and Elderly Care Medicine,

University Medical Center Groningen,

University of Groningen,

PO Box 196, 9700 AD Groningen,

The Netherlands.

Telephone: 0031 642637125

E-mail address: g.a.holtman@umcg.nl

For peer review only

For peer review only - http://bmjopen.bmj.com/site/about/guidelines.xhtml

ABSTRACT

Objectives: Patients who present in primary care with chronic functional somatic symptoms (FSS) have reduced quality of life and increased health care costs. Recognizing these early is a challenge. The aim is to develop and internally validate a clinical prediction rule for repeated consultations with FSS.

Design and setting: Records from the longitudinal population-based ("Lifelines") cohort study were linked to electronic health records from general practitioners (GPs).

Participants: We included patients consulting a GP with FSS within one year after baseline assessment in the Lifelines cohort.

Outcome measures: The outcome is repeated consultations with FSS, defined as ≥3 extra consultations for FSS within 1 year after the first consultation. Multivariable logistic regression, with bootstrapping for internal validation, was used to develop a risk prediction model from 14 literature-based predictors. Model discrimination, calibration, and diagnostic accuracy were assessed.

Results: 18,810 participants were identified by database linkage, of whom 2,650 consulted a GP with FSS and 297 (11%) had ≥3 extra consultations. In the final multivariable model, older age, female sex, lack of healthy activity, presence of generalized anxiety disorder, and higher number of GP consultations in the last year predicted repeated consultations. Discrimination after internal validation was 0.64 with a calibration slope of 0.95. The positive predictive value of patients with high scores on the model was 0.37 (0.29–0.47).

Conclusions: Several theoretically suggested predisposing and precipitating predictors, including neuroticism and stressful life events, surprisingly failed to contribute to our final model. Moreover, this model mostly included general predictors of increased risk of repeated consultations among patients with FSS. The model discrimination and positive predictive values were insufficient and preclude clinical implementation.

Keywords: medically unexplained symptoms, cohort studies, clinical decision rules, primary health care

For peer review only

Strengths and limitations of this study

- This study offers valuable insights into the predictors that could help general practitioners to identify repeated consultations with FSS.
- By linking routine health care data from primary care to a large population-based cohort, we could include relevant predictors based on epidemiological and theoretical factors from the literature and this approach may serve to enhance primary care research in the future.
- Each patient had a full follow-up of 1 year.
- Time from baseline assessment of the population-based cohort to first GP consultation varied, however, taking this variance into account did not affect the magnitude of the coefficients of the predictors in a substantial way, nor their selection.
- We did not externally validate the model, however the performance need to be improved before such research can be considered.

For peer review only

INTRODUCTION

Functional somatic symptoms (FSS), a synonymous of medically unexplained physical symptoms (MUPS), represent those that cannot be explained by a physical disease and account for about a third of all presentations in primary care,[1,2] clustering as cardiopulmonary, musculoskeletal, gastrointestinal, and general somatic symptoms.[3,4] However, these clusters appear to correlate and considered to represent one condition with different manifestations.[5] Most patients with FSS consult a general practitioner (GP) only once, but 10%–30% of cases will become chronic,[6] leading to more diagnostic tests, more referrals, higher health care costs, and more psychological distress compared with other patients.[7-9] Recognizing those patients at risk of developing chronic symptoms and consulted repeatedly the GP could therefore help to target interventions that reduce symptom severity,[10,11] improve quality of life, and reduce GP workloads. Ensuring that these patients are identified early is an important challenge facing GPs,[12] and one for which a validated clinical prediction rule may help. Several factors are known to increase the risk of chronicity of FSS, including predisposing (e.g., neuroticism), precipitating (e.g., physical and psychosocial stressors), and perpetuating (e.g., lack of healthy physical activity) factors.[13-15] Despite being described in the literature,[6] these factors have yet to be combined to predict repeated consultations with FSS in a clinical prediction rule for use in primary care.

In this study, we aimed to develop and internally validate a clinical prediction rule for repeated consultations among patients who consult GPs with FSS.

METHOD

Data sources

We linked patient records from the Lifelines Cohort Study (“Lifelines”)[16] with those from the Nivel Primary Care Database (NPCD).[17] Dutch law conditionally allows the use of such electronic health records for research purposes. Statistics Netherlands (CBS) then used temporary record

For peer review only - http://bmjopen.bmj.com/site/about/guidelines.xhtml

identification numbers to link records at an individual level for analysis.

Lifelines is a multidisciplinary prospective population-based cohort study using a three-generation design to examine the health and health-related behaviors of 167,729 people living in the north of the Netherlands.[16] It employs a broad range of investigative procedures to assess key factors that contribute to health and disease in the general population, focusing on multimorbidity and complex genetics. Lifelines was approved by the medical ethics committee of the University Medical Centre Groningen (2007/152) and was conducted in accordance with the Declaration of Helsinki. All participants signed an informed consent form.

The NPCD contains routinely recorded clinical data from GP consultations with patients, and is considered representative of the Dutch population.[17] The Dutch healthcare system is such that all non-institutionalized members of the population are registered with a general practice, which in turn, serves as a gatekeeping system through which patients must pass to access specialist care via GP referral.[18] In total, 528 general practices participated in 2019, and this study was approved according the Nivel Governance Code (number NZR0317.033).

For the current study, we included the baseline data of 152,728 adults enrolled in Lifelines between November 2006 and June 2013, and we linked these with the electronic health records of GP consultations for patients aged ≥18 years who consulted one of the 65 general practices in the north of the Netherlands that participated in the NPCD.

Patient population

We planned to include adults with FSS considered at risk of consulting the GP repeatedly, which we defined as those having a GP consultation for FSS in the year after their baseline assessment for Lifelines. The presence of FSS was assessed based on the International Classification of Primary Care (ICPC) codes that related to the symptoms that Robbins et al. described (Supplementary Table 1).[19]

Outcomes

For peer review only - http://bmjopen.bmj.com/site/about/guidelines.xhtml

The primary outcome was repeated consultations with FSS, defined as ≥3 extra GP consultations for one of the defined FSS (Supplementary Table 1) during a year of follow-up after first consulting a GP with that symptom.[19,20] Complete follow-up data were recorded for all GP consultations in electronic health records, and we permitted the FSS to vary between consultations.

Candidate predictors

We selected 14 predictors based on literature review and expert opinion: age, sex, neuroticism, chronic stress, stressful life events, self-rated health, healthy activity, body mass index (BMI), living alone, higher education, major depressive disorder (MDD), generalized anxiety disorder (GAD), and psychiatric or GP consultations in the 12 months before first consulting with FSS.[6,21] The data for these predictors were derived from the baseline of Lifelines, except for the psychiatric and GP consultations, which were derived from the NPCD.

Neuroticism was evaluated using an abridged version of the Neuroticism Extraversion Openness–Personality Inventory–Revised that included only anger-hostility, self-consciousness, impulsivity, and vulnerability, and excluded depression and anxiety (score range, 4–32).[22] Chronic stress was measured with the Long-term Difficulties Inventory (score range, 0–24).[23] The List of Threatening Events was used to assess the occurrence of 12 stressful life events (score range, 0–12).[23,24] Self-rated health was evaluated with the RAND-36 question[25] "how would you rate your health from 1 (excellent) to 5 (poor)." The Short Questionnaire to Assess Health-Enhancing Physical Activity was used to determine healthy activity behaviour, with a cut-off of 30 minutes at least 5 days a week indicating healthy activity.[26] Body weight and height were used to calculate BMI (weight (kg)/height (m^2)). Higher education was defined as at least secondary vocational education or work-based training. MDD and GAD were assessed by the Mini-International Neuropsychiatric Interview, compatible with International Classification of Disease, Tenth Edition, and the Diagnostic and Statistical Manual of Mental Disorders, Fourth Edition.[27] Psychiatric consultations was defined as patients with a consultation code in the P chapter of the ICPC and GP consultations was defined as

For peer review only

the number of total GP consultations in the 12 months before baseline of Lifelines.

Sample size

We estimated that we required 11,455 participants based on an assumption that 10% of the NPCD cohort would participate in Lifelines and that 75% of these data could be linked (i.e., 10% × 75% × 152,728). Given that the prevalence of repeated consultations with FSS has been reported to be 2.5%, we estimated that 286 of these could be included[20] to achieve an effective sample size of at least 20 outcome events per predictor.[28]

Missing data

Eleven predictors from Lifelines had missing data, so we evaluated the underlying causes and patterns to assess the conditions for multiple imputation.[29] We checked predictors of missingness and we assumed missing at random (MAR) when patients with missing values were different from patients without missing values with respect to observed variables. When data is MAR, we replaced all missing values by multiple imputation by chained equations (MICE), incorporating all variables used in the analyses, including the outcome variable, and all variables that predicted missingness of a certain variable or value. We imputed questionnaire sum scores rather than item scores. Finally, we constructed 20 imputed datasets combined across all datasets, pooled β coefficients, and calculated odds ratios using Rubin's rule.[30]

Statistical analysis

Repeated consultations with FSS over a one-year follow-up period was set as the binary outcome variable and associated with potential predictors as independent variables in logistic regression analyses. We performed univariable analyses to calculate unadjusted odds ratios.

To develop the clinical prediction rule, we initially included all potential predictors in a multivariable logistic regression model, irrespective of their univariable association and refrained from univariable preselection of candidate predictors to prevent model instability.[31] Using backward stepwise

For peer review only - http://bmjopen.bmj.com/site/about/guidelines.xhtml

selection, we excluded predictors from the model that were not statistically significant according to Akaike's information criterion (i.e., $p > 0.157$) in >50% of all imputed datasets.[32] Time in days between baseline assessment of predictors and first consultation differed between participants, so we also evaluated its influence in a separate analysis. We assessed rule performance by its discriminatory power with the C statistic and the calibration slope. We internally validated the model to correct for over-optimism by bootstrapping 250 samples, calculating a shrinkage factor, multiplying the original β coefficients by this factor, and re-estimating the intercepts using the shrunken β coefficients. The β coefficients were translated into a risk score of whole numbers for ease of use by GPs when evaluating the risk of repeated consultations in clinical practice. To that end, each β coefficient was divided by the coefficient closest to zero and then rounded to the nearest integer. The total score for each patient was calculated as the sum of all points for each predictor. We calculated the sensitivity, specificity, and positive predictive value of the rule at several thresholds to distinguish high and low risk. Thresholds were chosen arbitrarily based on the sample sizes being adequate in each category and the clinical risk being distinguishable.

All statistical analyses were performed with STATA/SE15 (STATA Corp, College station, TX, USA) and R (for bootstrapping). The Transparent Reporting of a multivariable prediction model for Individual Prediction of Diagnosis (TRIPOD) was used to conduct this study and report its results.[33]

Patient and public Involvement

Lifelines has a participant advisory board of eight active members with different background since 2016. The concept of this study was discussed during a meeting with this board. All Lifelines participants will receive the results of the study via a newsletter.

RESULTS

Study participants

For peer review only - http://bmjopen.bmj.com/site/about/guidelines.xhtml

Of the 152,728 Lifelines participants with a baseline assessment, we linked 18,810 (12%) with NPCD data (Figure 1). Among these, we included 2,650 participants (14% of those linked) attending GP consultations for FSS (i.e., the at-risk group), of whom 297 (11%) had ≥3 further consultations for FSS (i.e., the outcome criterion). The details of the included and excluded patients are summarized in Table 1, showing that the groups were broadly comparable. Notably, 24% of participants had a missing value and 3% had missing values for >4 predictors. The participants with missing values were slightly older and less active, and they less often had completed higher education (Supplementary Table 2).

Clinical rule development

Univariable associations of the potential predictors for repeated consultations with FSS are listed in Table 2. In the final multivariable model, the following five predictors were selected based on increasing the risk of repeated consultations: higher age, female sex, lack of healthy activity, presence of GAD, and having had more GP consultations in the year before first consulting with FSS (Table 3). Adjustment for time from baseline to first consultation did not affect the magnitude of the coefficients of the predictors in a substantial way, nor their selection. The shrinkage factor of 0.95 showed limited model overfitting and was applied to adjust predictor coefficients in the final model. Likewise, the C statistic (area under the curve) of 0.65 (95% CI, 0.62–0.69) was corrected to 0.64 (95% CI, 0.61–0.68). Agreement between the observed and predicted proportion of events showed adequate calibration (Supplementary Figure 1).

The final model could calculate the absolute predicted individual risk of repeated consultations with FSS (Supplementary Figure 2). For a risk score ≥100, the positive predictive value of repeated consultations was 0.37 (95% CI, 0.29–0.47) (Tables 4 and 5). However, when increasing the cut-off from 25 to 100, the sensitivity decreased from 0.87 (95% CI, 0.83–0.91) to 0.13 (95% CI, 0.10–0.17) and the specificity increased from 0.23 (95% CI, 0.22–0.25) to 0.97 (95% CI, 0.97–0.98).

For peer review only - http://bmjopen.bmj.com/site/about/guidelines.xhtml

DISCUSSION

Summary

We developed and internally validated a clinical prediction rule to identify patients at high risk of repeated consultations with FSS. This was based on five factors that are readily available in primary care: age, sex, activity levels, GAD diagnosis, and number of consultations. However, despite being well calibrated, the prediction rule showed poor discrimination. Nevertheless, if patients scored ≥100, the risk of repeated consultations with FSS increased to 37% from the baseline value of 11%.

Strengths and limitations

The study benefited from the use of a rich data set established by linking routine electronic health record data from primary care to a large population-based cohort. We effectively linked 18,810 patients (12%) from Lifelines who had at least one GP consultation, and we could include predictors based on epidemiological and theoretical factors from the literature, such as neuroticism and stressful life events.[21] The data linkage approach that we adopted may serve to enhance primary care research in the future. Each patient also had follow-up data for a full year, and although the time from baseline assessment in Lifelines to first GP consultation varied because of the dynamic nature of the NPCD cohort, this did not affect the results. Another strength is that we included 21 events per variable, resulting in minimal overfitting with a shrinkage factor of 0.95. An advantage of using dichotomous over continuous outcomes is that clinical interpretation is more straightforward. Although it is problematic that we did not externally validate the model, we contend that the model's performance will need to be improved before such research can be considered.

Our model predicts the risk of having ≥3 extra consultations for FSS. A developing underlying somatic disease could be suggested to ultimately explain some of these symptoms, however, a meta-analysis suggested that this risk is very low, reporting only 0.5% new diagnoses in follow-up studies of FSS.[34] FSS should not be confused with predicting a somatic symptom disorder or functional somatic syndrome, not least because we could not determine these diagnoses with the available data. In

For peer review only - http://bmjopen.bmj.com/site/about/guidelines.xhtml

addition, about 80% of patients with functional somatic syndrome will be missed using GP medical files.[35] A disadvantage of our outcome measure is that patients with FSS may also have consulted other health care professionals (e.g., physiotherapist), so these cases may have been missed. Therefore, the interpretation of our model is only applicable for GP consultations. We chose to use a follow-up of 1 year as this is often used in previous studies,[20,36] however persistent frequent attenders in primary care have more often FSS.[37] Therefore, a clinical prediction rule for repeated consultations with FSS during a longer follow-up might perform better. To avoid confusing and misunderstanding, we used the more neutral outcome of repeated consultations because our data did not allow for the identification of frequent attenders as defined in present day literature. The latter requires a comprehensive description of the contacts counted, e.g. how many were out-of-hours contacts, and how many were administrative or preventive consultations.[36] As we did not want to include too many predictors per variable to prevent overfitting, we a priori choose which predictors were relevant and feasible to use in a primary care setting. By this arbitrary selection, we may have missed relevant predictors (e.g. panic disorder and number of physical symptoms) that could have improved the performance of our prediction rule.[6,38] Our approach to identify the at-risk population first may explain the contrast with existing data. For example, we showed that 11% of patients presenting with FSS ultimately had ≥4 consultations for these symptoms, whereas previous research has shown a rate of 2.5% among all patients with GP consultations.[20]

Comparison with other studies

We are aware of no other clinical prediction rules for repeated GP consultations with FSS. It should be emphasized that such a model cannot be considered synonymous with explaining the cause.[39] However, we found three studies that developed models for persistent FSS by combining predictors using a backward or forward selection procedure. We limit our discussion to the three studies that developed a clinical prediction rule.

The first study used information from GP letters to medical specialists for patients who were

For peer review only - http://bmjopen.bmj.com/site/about/guidelines.xhtml

For peer review only

referred with FSS.[40] In their clinical prediction rule, female sex, referral symptom group, lack of somatic comorbidity, lack of abnormal physical findings, history of psychiatric diagnosis or treatment, and referral letter written in illness terminology were all shown to be predictors for FSS. This model had a higher area under the curve (0.80) than ours (0.64) and was developed for patients consulting internists. However, the GP referral letters included relevant predictors that helped to identify FSS, and although the population was more selected than ours, the results show that data collected in primary care can be suitable predictors.

The second study showed that the use of routine health care could include relevant predictors and developed a clinical prediction rule that potentially could be used to identify patients at risk for persistent FSS from routine primary care medical records.[41] The model had an area under the curve of 0.70 and the most important discriminative variable for persistent FSS was number of episodes. Just like our model they also included the predictors age, sex, and number of contacts.

The third study developed a model for symptom severity and for both physical and mental functioning during a 2-year follow-up period among patients with persistent FSS.[21] They predicted severe courses by physical comorbidity, higher baseline severity and longer physical symptom duration, anxiety, catastrophizing cognitions, embarrassment, and neuroticism, as well as fear avoidance, avoidance, or resting behaviour. By contrast, they predicted favourable courses based on limited alcohol use, higher education, higher baseline physical and mental functioning, symptom focusing, damage cognitions, and extraversion. Although we also identified anxiety as a predictor, we did not find the same for neuroticism or higher education. Also contrasting with our data, as well as that of others,[42,43] they did not show that female sex was a predictor. Unfortunately, we could not include predictors of illness behaviour because these were not evaluated in Lifelines. Indeed, the Symptoms Checklist 90 questionnaire had more than 50% missing values during baseline evaluation in Lifelines, so we excluded these data.[16] The differences in identified predictors may be explained by different study populations, predictor selection criteria, or outcomes.

For peer review only

Implications for research and practice

To our surprise, several theoretically suggested predisposing and precipitating predictors, including neuroticism and stressful life events, failed to contribute to the final prediction model. Instead, this model included mostly general predictors that provide little additional information to help GPs recognize patients at risk of consulting repeatedly with FSS, and it not only has poor discrimination and positive predictive value but also lacks external validation. Therefore, at present, we cannot recommend the score for clinical use. Nevertheless, our findings indicate that GPs might expect chronicity when older women with low activity levels and anxiety symptoms present with FSS. These require extra vigilance and may benefit from early intervention with self-help advice.[11] Some predictors identified in earlier studies, such as female sex and anxiety, could be potential factors in future clinical prediction rules designed to help GPs recognize patients at risk of consulting the GP repeatedly.

For peer review only

FUNDING

This study was funded by a grant from the Healthy Ageing Pilot of the University Medical Center Groningen (CDO17.0002/2017-1/296). The Lifelines initiative has been made possible by support from the Dutch Ministry of Health, Welfare and Sport; the Dutch Ministry of Economic Affairs; University Medical Center Groningen; Groningen University; and the Provinces in the north of the Netherlands (Drenthe, Friesland, Groningen).

ETHICAL APPROVAL

The medical ethics committee of the University Medical Centre Groningen approved the Lifelines study (2007/152). Dutch law conditionally allows the use of electronic health records for research purposes.

COMPETING INTERESTS

None declared.

PATIENT CONSENT FORM

Not required.

AUTHOR CONTRIBUTORS:

Study concept and design: GAH, HB, PFMV. Acquisition of data: PFMV and RAV. Analysis of data: GAH. Interpretation of data: GAH, HB, RAV, HW, MYB, JGMR, PFMV. Drafting the work: GAH, HB, PFMV. The final manuscript was critically revised and approved by all authors.

DATA AVAILABILITY STATEMENT:

The data used is part of two ongoing databases: Lifelines Cohort Study and Nivel Primary Care Database. The data is not available. The corresponding author can be contacted for details.

WORD COUNT

3024 words

For peer review only

ACKNOWLEDGMENT

We thank Dr Robert Sykes (www.doctored.org.uk) for providing editorial services.

For peer review only

REFERENCES

1. Jackson JL, Passamonti M. The outcomes among patients presenting in primary care with a physical symptom at 5 years. *Journal of general internal medicine*. 2005;20(11):1032-1037.

2. Rosendal M, Carlsen AH, Rask MT, Moth G. Symptoms as the main problem in primary care: A cross-sectional study of frequency and characteristics. *Scand J Prim Health Care*. 2015;33(2):91-99.

3. Fink P, Toft T, Hansen MS, Ornbol E, Olesen F. Symptoms and syndromes of bodily distress: An exploratory study of 978 internal medical, neurological, and primary care patients. *Psychosom Med*. 2007;69(1):30-39.

4. Rosmalen J, Tak L, De Jonge P. Empirical foundations for the diagnosis of somatization: Implications for DSM-5. *Psychol Med*. 2011;41(6):1133-1142.

5. Henningsen P, Zipfel S, Sattel H, Creed F. Management of functional somatic syndromes and bodily distress. *Psychother Psychosom*. 2018;87(1):12-31.

6. olde Hartman TC, Borghuis MS, Lucassen PL, van de Laar, Floris A, Speckens AE, van Weel C. Medically unexplained symptoms, somatisation disorder and hypochondriasis: Course and prognosis. A systematic review. *J Psychosom Res*. 2009;66(5):363-377.

7. Dirkzwager AJ, Verhaak PF. Patients with persistent medically unexplained symptoms in general practice: Characteristics and quality of care. *BMC Family Practice*. 2007;8(1):33.

8. Burton C, McGorm K, Richardson G, Weller D, Sharpe M. Healthcare costs incurred by patients repeatedly referred to secondary medical care with medically unexplained symptoms: A cost of illness study. *J Psychosom Res*. 2012;72(3):242-247.

For peer review only - http://bmjopen.bmj.com/site/about/guidelines.xhtml

9. De Waal, Margot W M, Arnold IA, Eekhof JA, Van Hemert AM. Somatoform disorders in general practice: Prevalence, functional impairment and comorbidity with anxiety and depressive disorders. *The British Journal of Psychiatry*. 2004;184(6):470-476.

10. Van Dessel N, Den Boeft M, van der Wouden, Johannes C, et al. Nonâ€pharmacological interventions for somatoform disorders and medically unexplained physical symptoms (MUPS) in adults. *Cochrane Database of Systematic Reviews*. 2014(11).

11. van Gils A, Schoevers RA, Bonvanie IJ, Gelauff JM, Roest AM, Rosmalen JG. Self-help for medically unexplained symptoms: A systematic review and meta-analysis. *Psychosom Med*. 2016;78(6):728-739.

12. Rosendal M, Hartman TCO, Aamland A, et al. â€œMedically unexplainedâ€ symptoms and symptom disorders in primary care: Prognosis-based recognition and classification. *BMC family practice*. 2017;18(1):18.

13. Frølund Pedersen H, Frostholm L, Søndergaard Jensen J, Ørnbøl E, Schröder A. Neuroticism and maladaptive coping in patients with functional somatic syndromes. *British journal of health psychology*. 2016;21(4):917-936.

14. Salit IE. Precipitating factors for the chronic fatigue syndrome. *J Psychiatr Res*. 1997;31(1):59-65.

15. Van Houdenhove B, Luyten P. Customizing treatment of chronic fatigue syndrome and fibromyalgia: The role of perpetuating factors. *Psychosomatics*. 2008;49(6):470-477.

16. Scholtens S, Smidt N, Swertz MA, et al. Cohort profile: LifeLines, a three-generation cohort study and biobank. *Int J Epidemiol*. 2014;44(4):1172-1180.

17. NIVEL. Https://Www.nivel.nl/nl/nivel-zorgregistraties-eerste-lijn/nivel-zorgregistraties-eerste-lijn. https://www.nivel.nl/nl/nivel-zorgregistraties-eerste-lijn/nivel-zorgregistraties-eerste-lijn.

For peer review only

18. Linden M, Gothe H, Ormel J. Pathways to care and psychological problems of general practice patients in a "gate keeper" and an" open access" health care system. *Soc Psychiatry Psychiatr Epidemiol*. 2003;38(12):690-697.

19. Robbins JM, Kirmayer LJ, Hemami S. Latent variable models of functional somatic distress. *J Nerv Ment Dis*. 1997;185(10):606-615.

20. Verhaak PF, Meijer SA, Visser AP, Wolters G. Persistent presentation of medically unexplained symptoms in general practice. *Fam Pract*. 2006;23(4):414-420.

21. Claassen-van Dessel N, van der Wouden, Johannes C, Twisk JW, Dekker J, van der Horst, Henriëtte E. Predicting the course of persistent physical symptoms: Development and internal validation of prediction models for symptom severity and functional status during 2 years of follow-up. *J Psychosom Res*. 2018;108:1-13.

22. Costa PT, Mac Crae RR. *Neo personality inventory-revised (NEO PI-R).* Psychological Assessment Resources Odessa, FL; 1992.

23. Rosmalen J, Bos E, De Jonge P. Validation of the long-term difficulties inventory (LDI) and the list of threatening experiences (LTE) as measures of stress in epidemiological population-based cohort studies. *Psychol Med*. 2012;42(12):2599-2608.

24. Brugha TS, Cragg D. The list of threatening experiences: The reliability and validity of a brief life events questionnaire. *Acta Psychiatr Scand*. 1990;82(1):77-81.

25. Hays RD, Morales LS. The RAND-36 measure of health-related quality of life. *Ann Med*. 2001;33(5):350-357.

For peer review only

26. Wendel-Vos GW, Schuit AJ, Saris WH, Kromhout D. Reproducibility and relative validity of the short questionnaire to assess health-enhancing physical activity. *J Clin Epidemiol*. 2003;56(12):1163-1169.

27. Sheehan DV, Lecrubier Y, Sheehan KH, et al. The mini-international neuropsychiatric interview (MINI): The development and validation of a structured diagnostic psychiatric interview for DSM-IV and ICD-10. *J Clin Psychiatry*. 1998.

28. Peduzzi P, Concato J, Kemper E, Holford TR, Feinstein AR. A simulation study of the number of events per variable in logistic regression analysis. *J Clin Epidemiol*. 1996;49(12):1373-1379.

29. Hughes RA, Heron J, Sterne JA, Tilling K. Accounting for missing data in statistical analyses: Multiple imputation is not always the answer. *Int J Epidemiol*. 2019;1:11.

30. Marshall A, Altman DG, Holder RL, Royston P. Combining estimates of interest in prognostic modelling studies after multiple imputation: Current practice and guidelines. *BMC Med Res Methodol*. 2009;9:57-2288-9-57.

31. Harrell Jr FE, Lee KL, Mark DB. Multivariable prognostic models: Issues in developing models, evaluating assumptions and adequacy, and measuring and reducing errors. *Stat Med*. 1996;15(4):361-387.

32. Sauerbrei W. The use of resampling methods to simplify regression models in medical statistics. *Journal of the Royal Statistical Society: Series C (Applied Statistics)*. 1999;48(3):313-329.

33. Collins GS, Reitsma JB, Altman DG, Moons KG. Transparent reporting of a multivariable prediction model for individual prognosis or diagnosis (TRIPOD): The TRIPOD statement. *BMC medicine*. 2015;13(1):1.

For peer review only

34. Eikelboom E, Tak L, Roest A, Rosmalen J. A systematic review and meta-analysis of the percentage of revised diagnoses in functional somatic symptoms. *J Psychosom Res*. 2016;88:60-67.

35. den Boeft M, van der Wouden, Johannes C, Rydell-Lexmond TR, de Wit NJ, van der Horst, Henriëtte E, Numans ME. Identifying patients with medically unexplained physical symptoms in electronic medical records in primary care: A validation study. *BMC family practice*. 2014;15(1):109.

36. Vedsted P, Christensen MB. Frequent attenders in general practice care: A literature review with special reference to methodological considerations. *Public Health*. 2005;119(2):118-137.

37. Smits FT, Brouwer HJ, ter Riet G, van Weert HC. Epidemiology of frequent attenders: A 3-year historic cohort study comparing attendance, morbidity and prescriptions of one-year and persistent frequent attenders. *BMC Public Health*. 2009;9(1):36.

38. Smits FT, Brouwer HJ, Zwinderman AH, et al. Why do they keep coming back? psychosocial etiology of persistence of frequent attendance in primary care: A prospective cohort study. *J Psychosom Res*. 2014;77(6):492-503.

39. Moons KG, Royston P, Vergouwe Y, Grobbee DE, Altman DG. Prognosis and prognostic research: What, why, and how? *BMJ*. 2009;338:b375.

40. Gol JM, Burger H, Janssens KA, Slaets JP, Gans RO, Rosmalen JG. PROFSS: A screening tool for early identification of functional somatic symptoms. *J Psychosom Res*. 2014;77(6):504-509.

41. den Boeft M. Medically unexplained physical symptoms in primary care: Identification, management and societal aspects. . 2016.

42. Speckens A, Van Hemert A, Bolk J, Rooijmans H, Hengeveld M. Unexplained physical symptoms: Outcome, utilization of medical care and associated factors. *Psychol Med*. 1996;26(4):745-752.

For peer review only - http://bmjopen.bmj.com/site/about/guidelines.xhtml

43. Leiknes KA, Finset A, Moum T, Sandanger I. Methodological issues concerning lifetime medically unexplained and medically explained symptoms of the composite international diagnostic interview: A prospective 11-year follow-up study. *J Psychosom Res*. 2006;61(2):169-179.

For peer review only

For peer review only - http://bmjopen.bmj.com/site/about/guidelines.xhtml

Figure 1. Patient selection flowchart

Note: extra consultations (≥3) refers to additional presentations for FSS during a one-year follow-up period after an initial GP consultation for FSS.

For peer review only

For peer review only - http://bmjopen.bmj.com/site/about/guidelines.xhtml

Table 1. Characteristics of included and excluded patients

	Included patients[a] (n = 2,650)		Excluded patients[b] (n = 7,418)	
	N (%)	Score	*n*	Score
Age, mean y (SD)	2,636 (99)	45 (14)	7,387	45 (14)
Female, n (%)	2,650 (100)	1,802 (68)	7,418	4,577 (62)
Neuroticism, median (IQR)	2,248 (85)	10.1 (9.1-11.3)	6,419	9.9 (8.9 – 11)
Chronic stress, median (IQR)	2,465 (93)	2 (1–4)	7,005	2 (1–4)
Stressful life events, median (IQR)	2,464 (93)	1 (0–2)	7,008	1 (0–2)
Self-rated health, median (IQR)	2,548 (96)	3 (2–3)	7,228	3 (2–3)
Healthy activity[c], n (%)	2,274 (86)	1,259 (55)	6,505	3,589 (55)
Body mass index (kg/m^2), median (IQR)	2,648 (100)	26 (23–28)	7,417	25 (23–28)
Living alone, n (%)	2,522 (95)	335 (13.3)	7,167	904 (12.6)
Higher education[d], n (%)	2,572 (97)	1,707 (66)	7,215	5,067 (70)
MDD[f], n (%)	2,555 (96)	86 (3)	7,248	176 (2.4)
GAD[g], n (%)	2,555 (96)	165 (6)	7,248	351 (4.8)
Psychiatric consultations last year[h,i], n (%)	2,650 (100)	292 (11)	7,418	783 (11)
GP consultations last year[i], median (IQR)	2,650 (100)	2 (0–5)	7,418	1 (0–3)

[a] Included: GP consultations and ≥1 FSS within 1 year after baseline Lifelines assessment.
[b] Excluded: GP consultation without FSS within 1 year after baseline of Lifelines.
[c] Healthy activity, defined as 30 minutes at least 5 days a week.
[d] Higher education, defined as at least secondary vocational education or work-based training.
[f] MDD: Major Depressive Disorder.
[g] GAD: Generalized Anxiety Disorder.
[h] Patients with a consultation code in the P chapter of the International Classification of Primary Care.
[i] Predictors from NPCD. Other predictors are from Lifelines.
Abbreviations: SD, standard deviation; IQR, Interquartile Range; GP, general practitioner

For peer review only

Table 2. Univariable analysis of predictors for repeated consultations with FSS

Variable	Repeated consultations[a] OR (95% CI)
Age	1.01 (1.00–1.02)
Sex (m)	0.69 (0.52–0.91)
Neuroticism	1.08 (0.99–1.17)
Chronic stress	1.04 (0.99–1.09)
Stressful life events	1.08 (0.99–1.18)
Self-rated health	1.41 (1.20–1.66)
Healthy activity[b]	0.70 (0.53–0.91)
Body mass index (kg/m^2)	1.04 (1.01–1.07)
Living alone	0.91 (0.62–1.32)
Higher education[c]	0.75 (0.58–0.96)
MDD[d]	1.53 (0.85–2.73)
GAD[e]	2.15 (1.45–3.19)
Psychiatric consultations last year[f,g]	1.17 (1.04–1.33)
GP consultations last year[g]	1.12 (1.09–1.15)

[a] Outcome, ≥3 extra FSS consultations during a 1-year follow-up period (n = 297).
[b] Healthy activity, defined as 30 minutes at least 5 days a week.
[c] Higher education, defined as at least secondary vocational education or work-based training.
[d] MDD: Major Depressive Disorder.
[e] GAD: Generalized Anxiety Disorder.
[f] Number of consultations concerning International Classification of Primary Care codes in the P chapter.
[g] Predictors are from NPCD and are continuous. Other predictors are from the Lifelines database.
Abbreviations: CI, confidence interval; GP, general practitioner

For peer review only

Table 3. Final multivariable analysis for repeated consultations with FSS

Predictors	OR (95% CI) [c]	P-value	Coefficient	Adjusted coefficient	Risk score
Constant	0.05 (0.03–0.08)	0.000	-2.95	-3.80	
Age	1.02 (1.01–1.03)	0.000	0.02	0.02	1
Sex (m)	0.75 (0.56–0.99)	0.042	-0.30	-0.29	-15
Healthy activity[a]	0.60 (0.45–0.80)	0.001	-0.51	-0.48	-24
GAD[b]	1.79 (1.17–2.74)	0.008	0.58	0.56	28
GP consultations last year	1.10 (1.07–1.14)	0.000	0.10	0.10	5

[a] Healthy activity, defined as 30 minutes at least 5 days a week.
[b] GAD: Generalized Anxiety Disorder.
[c] OR (95% CI): Odds Ratio (95% confidence interval).
Note: Shrinkage factor 0.96; predictors selected if P < 0.157.
Abbreviations: CI, confidence interval; GP, general practitioner

For peer review only

Table 4. Risk of repeated consultations with FSS by different cut-off scores

Cut-off score	*n*	Outcome	Observed risk	Predicted risk
<25	585	38	0.07	0.06
25–49	1009	82	0.08	0.08
50–99	952	138	0.15	0.14
≥100	104	39	0.38	0.37

Note: the risk score was calculated by multiplying each risk score by the predictor value, with the total score ranging from −21 to 301 for all included patients (for example -21 represents the following patient: 18 years (18), male (-15), healthy activity (-24), no GAD (0), no GP consultation last year (0) (=18-15-24+0+0=-21) and 301: 63 years (63), female (0), lack of healthy activity (0), presence of GAD (28), 42 GP consultations last year (210) (=63+0+0+28+210=301)).

For peer review only

Table 5. Diagnostic accuracy of the risk score for repeated consultations with FSS

Cut-off score	*n*	Sensitivity (95% CI) [c]	Specificity (95% CI) [c]	PPV (95% CI)	NPV (95% CI)
≥25	2065	0.87 (0.83–0.91)	0.23 (0.22–0.25)	0.13 (0.11–0.14)	0.93 (0.91–0.95)
≥50	1057	0.59 (0.54–0.65)	0.63 (0.61–0.64)	0.17 (0.15–0.19)	0.92 (0.91–0.94)
≥100	104	0.13 (0.10–0.17)	0.97 (0.97–0.98)	0.37 (0.29–0.47)	0.91 (0.90–0.92)

Abbreviations: PPV, positive predictive value; NPV, negative predictive value, CI, confidence interval.

For peer review only

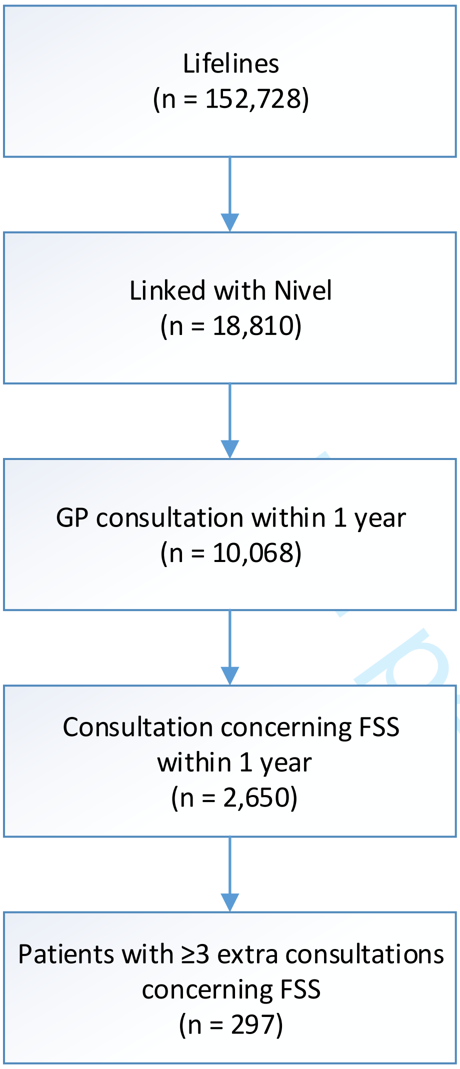

For peer review only

Supplementary Table 1. Functional somatic symptoms and the corresponding International Classification of Primary Care codes

Symptom	International Classification of Primary Care code
Generalized pain	A01
Fatigue/Tiredness/weakness	A04
Abdominal pain	D01
Flatulence	D08
Nauseous	D09
Constipation	D12
Defecation problems	D18
Irritable Bowel Syndrome	D93
Chest pain	K01; K02
Palpitations	K04
Joint pain	L01; L08
Back pain	L02; L03
Extremities pain	L09; L14
Headache	N01; N02
Wheezy	N02
Dizzy	N20
Sleep disorder	P06
Concentration problems	P20
Sore Throat	R21
Loss in appetite	T03
Weight gain/loss	T07; T08

For peer review only

For peer review only - http://bmjopen.bmj.com/site/about/guidelines.xhtml

Supplementary Table 2. Comparison of patients with and without missing values

	No missing values (n = 2,002)		Missing values (n = 648)		Univariable OR (95% CI)
	n	value	*n*	value	
Age, mean y (SD)	2,002	42 (12)	634	54 (17)	**1.07 (1.06–1.07)**
Male, n (%)	2,002	622 (31)	648	226 (35)	1.19 (0.99–1.43)
Neuroticism, median (IQR)	2,002	10.1 (9.1–11.3)	246	10.3 (9.3–11.5)	1.04 (0.96–1.12)
Chronic stress, median (IQR)	2,002	3 (1–4)	463	2 (0–4)	**0.90 (0.86–0.94)**
Stressful life events, median (IQR)	2,002	1 (0–2)	462	1 (0–2)	**1.09 (1.02–1.17)**
Self-rated health, median (IQR)	2,002	3 (2–3)	546	3 (2–3)	1.10 (0.97–1.25)
Healthy activity[a], n (%)	2,002	1,056 (53)	272	203 (75)	**2.63 (1.98–3.51)**
Body mass index (kg/m^2), median (IQR)	2,002	25 (23–28)	646	26 (24–29)	**1.05 (1.03–1.07)**
Living alone, n (%)	2,002	233 (12)	520	102 (20)	**1.85 (1.43–2.39)**
Higher education[b], n (%)	2,002	1,412 (71)	570	295 (52)	**0.45 (0.37–0.54)**
MDD[c], n (%)	2,002	64 (3)	553	22 (4)	1.25 (0.77–2.06)
GAD[d], n (%)	2,002	138 (7)	553	27 (5)	0.69 (0.45–1.06)
Psychiatric consultations last year[e,f], n (%)	2,002	205 (10)	648	87 (13)	1.07 (0.96–1.19)
GP consultations last year[f], median (IQR)	2,002	2 (0–5)	648	3 (1–6)	**1.05 (1.03–1.08)**
Repeated consultations[g], n (%)	2,002	200 (10)	648	97 (15)	**1.59 (1.22–2.06)**

[a] Healthy activity, defined as 30 minutes at least 5 days a week.
[b] Higher education, defined as at least secondary vocational education or work-based training.
[c] MDD: Major Depressive Disorder.
[d] GAD: Generalized Anxiety Disorder.
[e] Patients with a consultation code in the P chapter of the International Classification of Primary Care.
[f] Predictors from NPCD and both are continuous variables. Other predictors are from Lifelines database.
[g] Repeated consultations is defined as ≥3 extra functional somatic symptoms consultations during one year of follow-up.
Note: 24% (648/2,650) had a missing value
Abbreviations: SD, standard deviation; IQR, Interquartile Range; GP, general practitioner; OR, odds ratio

For peer review only

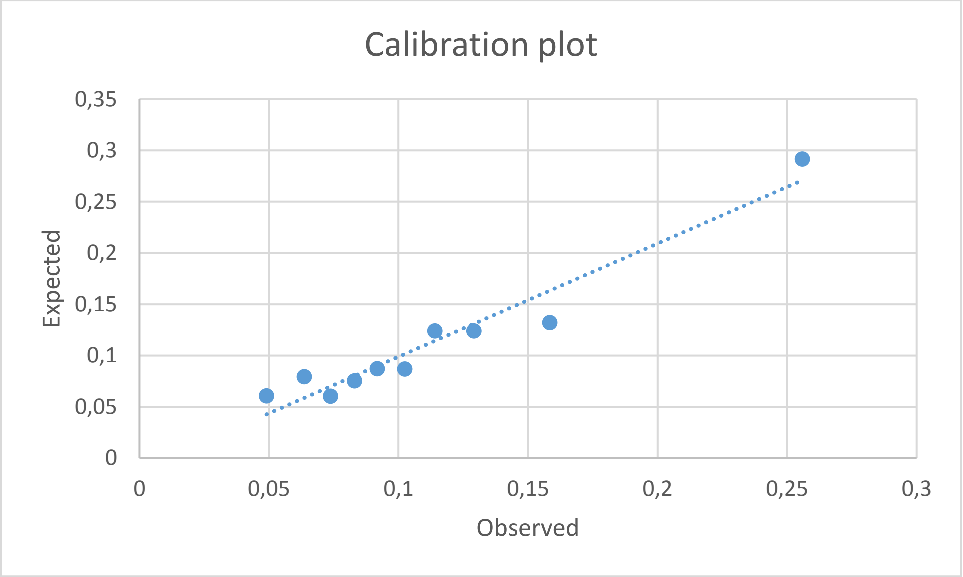

Supplementary Figure 1. Calibration plot of the clinical prediction rule for repeated consultations with functional somatic symptoms

For peer review only - http://bmjopen.bmj.com/site/about/guidelines.xhtml

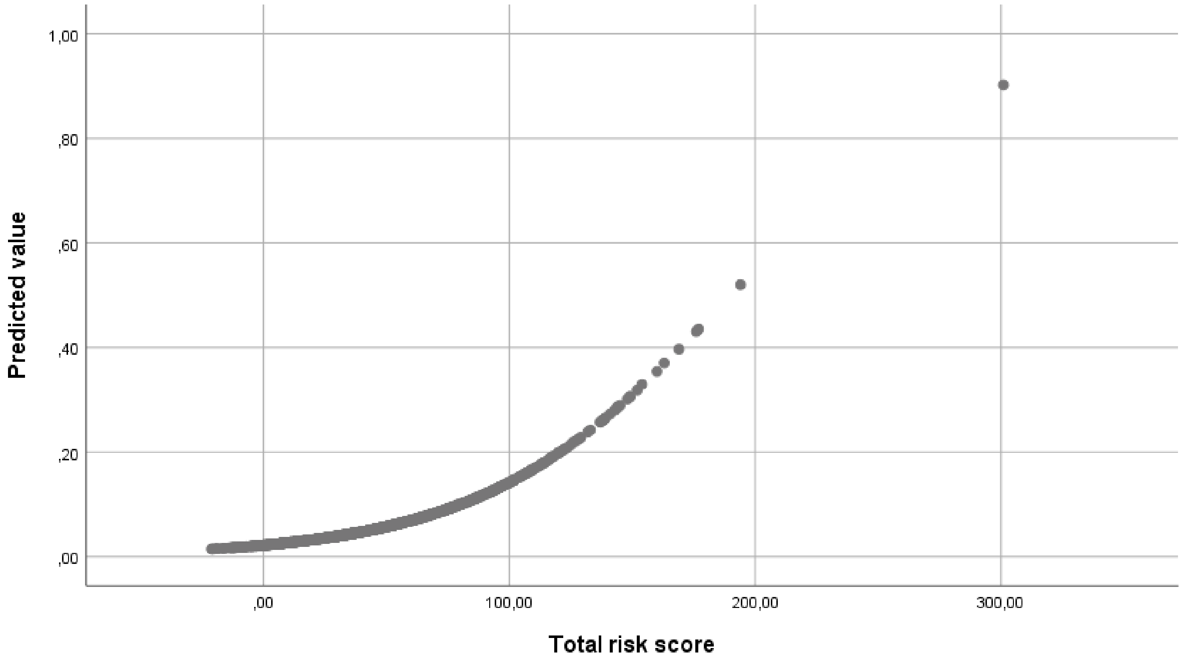

Supplemental Figure 2. Relation between the total risk score and the predicted risk of repeated consultations with functional somatic symptoms

For peer review only - http://bmjopen.bmj.com/site/about/guidelines.xhtml

TRIPOD Checklist: Prediction Model Development

Section/Topic	Item	Checklist Item	Page
**Title and abstract**			
Title	1	Identify the study as developing and/or validating a multivariable prediction model, the target population, and the outcome to be predicted.	1
Abstract	2	Provide a summary of objectives, study design, setting, participants, sample size, predictors, outcome, statistical analysis, results, and conclusions.	2
**Introduction**			
Background and objectives	3a	Explain the medical context (including whether diagnostic or prognostic) and rationale for developing or validating the multivariable prediction model, including references to existing models.	3
	3b	Specify the objectives, including whether the study describes the development or validation of the model or both.	3
**Methods**			
Source of data	4a	Describe the study design or source of data (e.g., randomized trial, cohort, or registry data), separately for the development and validation data sets, if applicable.	4,5
	4b	Specify the key study dates, including start of accrual; end of accrual; and, if applicable, end of follow-up.	5,6
Participants	5a	Specify key elements of the study setting (e.g., primary care, secondary care, general population) including number and location of centres.	5
	5b	Describe eligibility criteria for participants.	5
	5c	Give details of treatments received, if relevant.	NA
Outcome	6a	Clearly define the outcome that is predicted by the prediction model, including how and when assessed.	5,6
	6b	Report any actions to blind assessment of the outcome to be predicted.	NA
Predictors	7a	Clearly define all predictors used in developing or validating the multivariable prediction model, including how and when they were measured.	6
	7b	Report any actions to blind assessment of predictors for the outcome and other predictors.	NA
Sample size	8	Explain how the study size was arrived at.	6,7
Missing data	9	Describe how missing data were handled (e.g., complete-case analysis, single imputation, multiple imputation) with details of any imputation method.	7
Statistical analysis methods	10a	Describe how predictors were handled in the analyses.	7
	10b	Specify type of model, all model-building procedures (including any predictor selection), and method for internal validation.	7,8
	10d	Specify all measures used to assess model performance and, if relevant, to compare multiple models.	7,8
Risk groups	11	Provide details on how risk groups were created, if done.	8
**Results**			
Participants	13a	Describe the flow of participants through the study, including the number of participants with and without the outcome and, if applicable, a summary of the follow-up time. A diagram may be helpful.	8
	13b	Describe the characteristics of the participants (basic demographics, clinical features, available predictors), including the number of participants with missing data for predictors and outcome.	8
Model development	14a	Specify the number of participants and outcome events in each analysis.	8
	14b	If done, report the unadjusted association between each candidate predictor and outcome.	8
Model specification	15a	Present the full prediction model to allow predictions for individuals (i.e., all regression coefficients, and model intercept or baseline survival at a given time point).	9
	15b	Explain how to the use the prediction model.	9
Model performance	16	Report performance measures (with CIs) for the prediction model.	9
**Discussion**			
Limitations	18	Discuss any limitations of the study (such as nonrepresentative sample, few events per predictor, missing data).	10
Interpretation	19b	Give an overall interpretation of the results, considering objectives, limitations, and results from similar studies, and other relevant evidence.	11
Implications	20	Discuss the potential clinical use of the model and implications for future research.	11,12
**Other information**			
Supplementary information	21	Provide information about the availability of supplementary resources, such as study protocol, Web calculator, and data sets.	13
Funding	22	Give the source of funding and the role of the funders for the present study.	13

We recommend using the TRIPOD Checklist in conjunction with the TRIPOD Explanation and Elaboration document.
